# Persistent COUP-TFII expression underlies the myopathy and impaired muscle regeneration observed in resistance to thyroid hormone-alpha

**DOI:** 10.1038/s41598-021-84080-5

**Published:** 2021-02-25

**Authors:** Paola Aguiari, Yan-Yun Liu, Astgik Petrosyan, Sheue-yann Cheng, Gregory A. Brent, Laura Perin, Anna Milanesi

**Affiliations:** 1 David Geffen School of Medicine at UCLA - VA Healthcare System, Los Angeles, CA USA; 2 GOFARR Laboratory for Organ Regenerative Research and Cell Therapeutics in Urology, Children’s Hospital Los Angeles, Los Angeles, CA USA; 3 National Cancer Institute, Bethesda, MD USA

**Keywords:** Muscle stem cells, Hormone receptors, Thyroid diseases

## Abstract

Thyroid hormone signaling plays an essential role in muscle development and function, in the maintenance of muscle mass, and in regeneration after injury, via activation of thyroid nuclear receptor alpha (THRA). A mouse model of resistance to thyroid hormone carrying a frame-shift mutation in the THRA gene (THRA-PV) is associated with accelerated skeletal muscle loss with aging and impaired regeneration after injury. The expression of nuclear orphan receptor chicken ovalbumin upstream promoter-factor II (COUP-TFII, or *Nr2f2*) persists during myogenic differentiation in THRA-PV myoblasts and skeletal muscle of aged THRA-PV mice and it is known to negatively regulate myogenesis. Here, we report that in murine myoblasts COUP-TFII interacts with THRA and modulates THRA binding to thyroid response elements (TREs). Silencing of COUP-TFII expression restores in vitro myogenic potential of THRA-PV myoblasts and shifts the mRNA expression profile closer to WT myoblasts. Moreover, COUP-TFII silencing reverses the transcriptomic profile of THRA-PV myoblasts and results in reactivation of pathways involved in muscle function and extracellular matrix remodeling/deposition. These findings indicate that the persistent COUP-TFII expression in THRA-PV mice is responsible for the abnormal muscle phenotype. In conclusion, COUP-TFII and THRA cooperate during post-natal myogenesis, and COUP-TFII is critical for the accelerated skeletal muscle loss with aging and impaired muscle regeneration after injury in THRA-PV mice.

## Introduction

Skeletal muscle is a major target of thyroid hormone signaling. Myopathic changes (including muscular dystrophy and weakness) are commonly described in hypothyroid and hyperthyroid patients^1,2^.

Triiodothyronine hormone (T3) role in embryonic muscle development, post-natal muscle growth, in maintaining skeletal muscle during adult life by regulating metabolism, muscle function and muscle regeneration is well documented^3–5^. The genomic actions of T3 are mediated by thyroid hormone nuclear receptors THRA (alpha) and THRB (beta), which act as ligand-inducible transcription factors^6^. THRA is the predominant isoform in skeletal muscle and plays an essential role in skeletal muscle regeneration after injury and maintenance of muscle mass with aging^7,8^. A mouse model carrying a frame-shift mutation in the THRA gene (THRA-PV) shows resistance to thyroid hormone (RTH) and presents abnormal skeletal muscle phenotype^8,9^. Myoblasts derived from THRA-PV mice show reduced proliferation and myogenic differentiation in in vitro models, and skeletal muscle of THRA-PV mice show impaired in vivo regeneration after cardiotoxin (CTX)- induced injury^7^. Moreover, THRA-PV mice have skeletal muscle loss and impairment in skeletal muscle regeneration with aging^8^.

Chicken ovalbumin upstream promoter-factor II (COUP-TFII, or *Nr2f2*) is a member of the nuclear orphan receptor superfamily. COUP-TFII is required for embryonic development, as *COUP-TFII*^*-/-*^ mice embryos are embryonically lethal due to defective heart development and angiogenesis^10^.

COUP-TFII expression has been detected in undifferentiated mesenchymal precursors, but not in mature cells, suggesting its role in maintaining stem cell function^11^. In mesenchymal stem cells, COUP-TFII acts as a switch among mesenchymal lineages promoting the development of cartilage and white adipocytes while blocking myogenesis, osteogenesis and brown adipose tissue specification^12^.

COUP-TFII is required for embryonic skeletal muscle development^13^. In skeletal muscle, COUP-TFII is expressed by satellite cells (SCs) and negatively regulates myogenesis by repression of myoblast fusion^14^. Overexpression of COUP-TFII in murine myoblast inhibits myogenesis through modulation of Myf5 and MyoD expression, and contributes to Duchenne-like Muscular Dystrophies^15^. Interestingly, a mouse model selectively expressing COUP-TFII in SCs was associated with a skeletal muscle phenotype similar to the one of THRA-PV mice, including smaller fibers with centrally located nuclei and defective regeneration after CTX-induced injury^15^. Moreover, COUP-TFII-overexpressing mice develop signs of progressive Duchenne-like myopathy with aging^15^.

Here we report that COUP-TFII interacts with THRA, and that this interaction mediates impaired skeletal muscle regeneration and skeletal muscle loss with aging in a mouse model of RTH.

## Results

### COUP-TFII expression persists during myogenic differentiation in THRA-PV myoblasts and in skeletal muscle of aged THRA-PV mice

**Figure 1:**
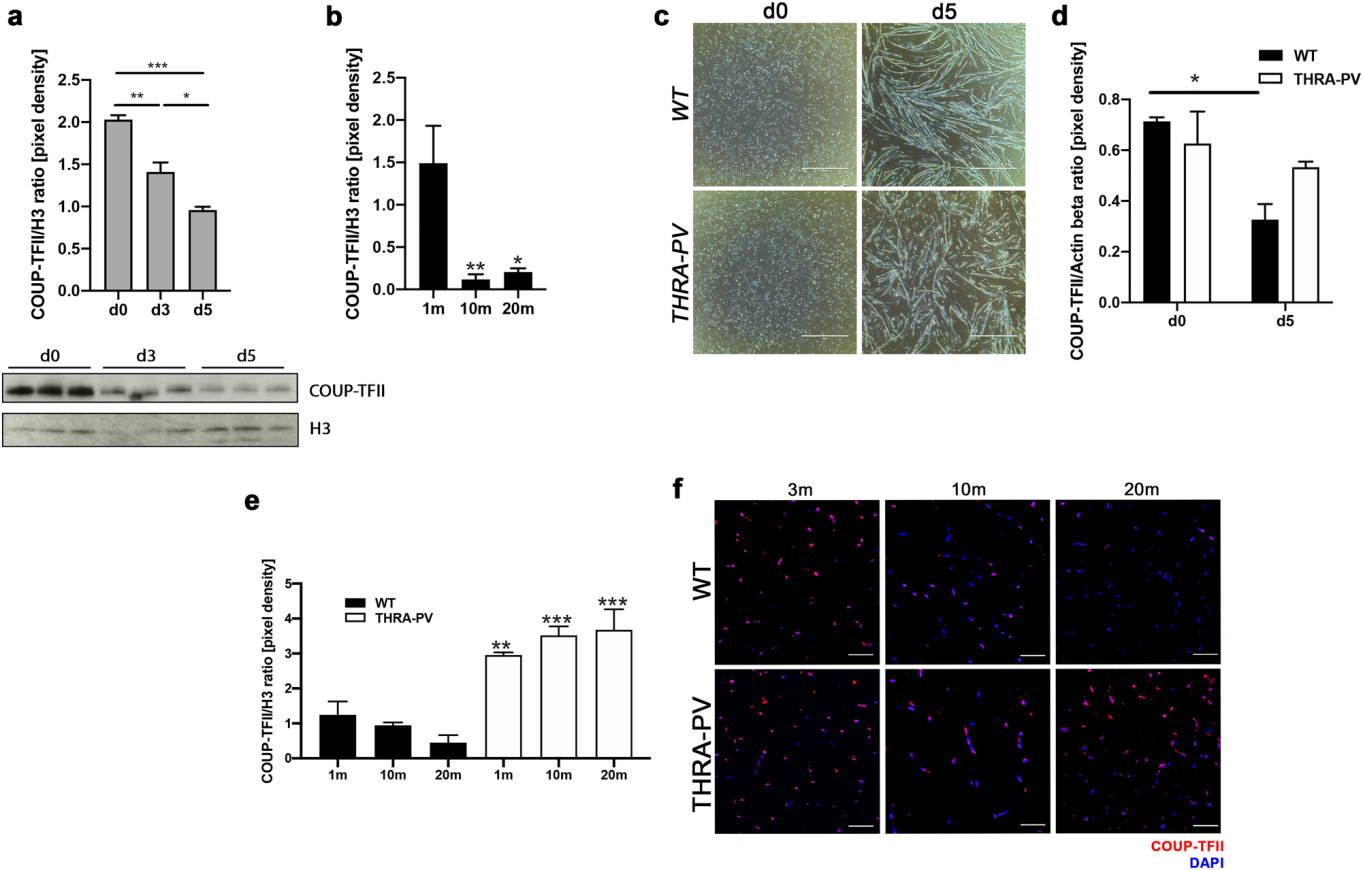
COUP-TFII expression during myogenic differentiation and skeletal muscle aging in THRA-PV mice. (**a**) Western blot of COUP-TFII expression in C2C12 myoblasts before (d0) and at 3 (d3) and 5 (d5) days of differentiation. Upper panel: quantification of the expression normalized to histone H3 (H3). Lower panel: crop of immunoblots of COUP-TFII (47 kDa) and H3 (17 kDa). Full-length gels are presented in Supplementary Figure S1. (**b**) Western blot analysis of COUP-TFII expression in skeletal muscle of WT mice at different ages (1, 10 and 20 months). (**c**) Phase contrast images of WT and THRA-PV myoblasts before (d0) and after 5 days of differentiation (d5). Scale bar = 1000 µm (**d**) Western Blot analysis of COUP-TFII expression in WT and THRA-PV myoblasts from before differentiation (d0) and five days post-differentiation (d5). (**e**) COUP-TFII protein expression in skeletal muscle of THRA-PV and control littermate mice during aging at 1, 10 and 20 months. (**f**) Immunofluorescence for COUP-TFII in WT and THRA-PV skeletal muscle at 3, 10 and 20 months. COUP-TFII in red, DAPI in blue. Scale bar = 25 µm. Data are shown as mean ± SEM, n = 3. One or two-way ANOVA: *p < 0.05, **p < 0.01, ***p < 0.001, in (**b**) compared to 1 month and (**d**) compared to age-matched control (WT).

First, we confirmed that COUP-TFII protein expression declines during myogenic differentiation in C2C12 myoblasts (Fig. 1a and S1), as previously reported^14^. A higher nuclear expression of COUP-TFII was detected in proliferating WT C2C12 myoblasts and declined significantly by day 5 of differentiation (d0 vs d3 *p* = 0.0026; d0 vs d5 *p* = 0.0001; d3 vs d5 *p* = 0.0120). We further examined the expression of COUP-TFII in the skeletal muscle of mice at different ages and noted a significant decline of its expression with age in both male and female mice (1 m vs. 10 m *p* = 0.0089, FC = −12.7; 1 m vs. 20 m *p* = 0.0142, FC = −7.3, Fig. 1b and S2).

To investigate the role of COUP-TFII in the skeletal muscle phenotype of the THRA-PV mice, we examined COUP-TFII expression during myogenic differentiation of THRA-PV-derived myoblasts and in aged skeletal muscle. Myoblasts from THRA-PV mice had impaired differentiation at day 5 in culture, compared with WT myoblasts (Fig. 1c). COUP-TFII expression decreased at day 5 of differentiation in WT myoblasts, as was seen in C2C12 cells (*p* = 0.0292, FC = −2.2, Fig. 1d and S3). However, in THRA-PV myoblasts, COUP-TFII expression remained high with no reduction during differentiation (Fig. 1d). Previously, we showed that skeletal muscle fibers of THRA-PV adult and older mice (age 10 and 20 months) are significantly smaller than the WT muscle fibers^8^. Young THRA-PV mice (3-month-old) also showed significantly smaller muscle fiber size compared with the WT mice (*p* < 0.0001, FC = −1.7, Supplementary Figure S4). COUP-TFII protein expression was higher in the skeletal muscle of younger WT mice (1 month) and progressively declined with age (20 months). In contrast, COUP-TFII expression in THRA-PV skeletal muscle persisted at a high level with aging (Fig. 1e, S5 and 1f), as demonstrated by both western blot (WT vs THRA-PV at 1 m *p* = 0.0125, FC = +2.4; at 10m* p* = 0.0004, FC = +3.7; at 20 m *p* = 0.0001, FC = +8.2, Fig. 1e, S5) and immunofluorescence (Fig. 1f).

These observations suggest that sustained expression of COUP-TFII might play a key role in the myogenic program of THRA-PV mice.

### COUP-TFII interacts with THRA and modulates its binding to TREs

We observed that THRA-PV myoblasts present a higher sustained expression of COUP-TFII during myogenic differentiation compared to WT myoblasts, suggesting an interplay between COUP-TFII and THRA.

**Figure 2:**
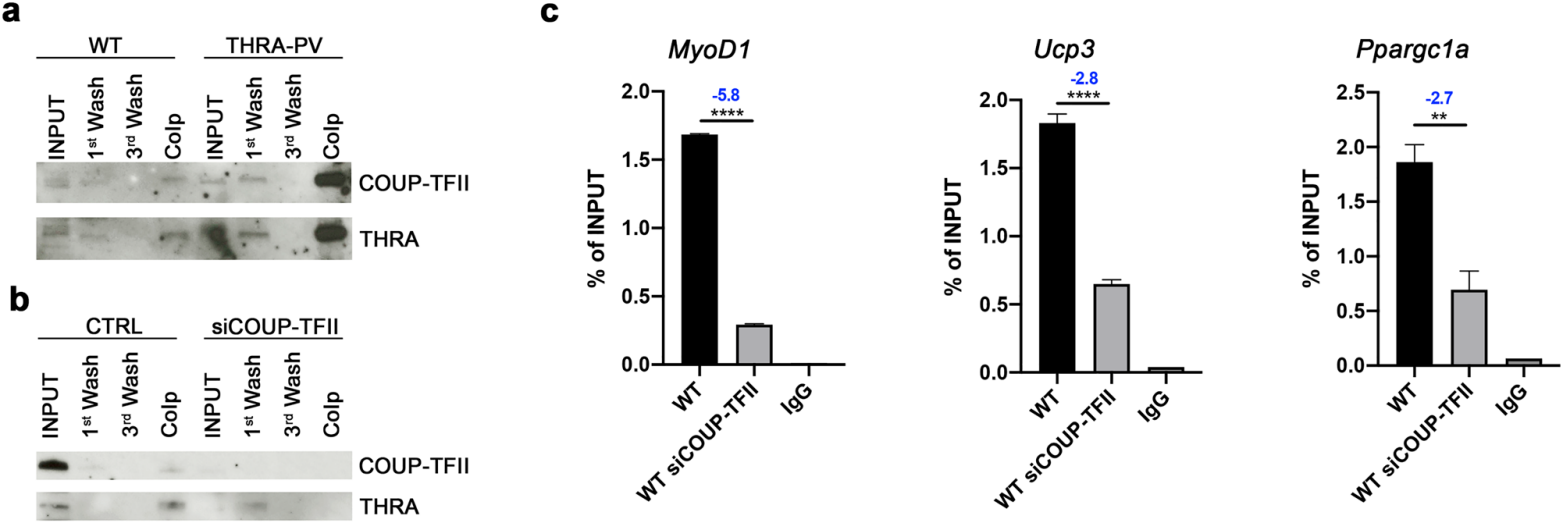
COUP-TFII interacts with THRA and modulates its binding to TREs. (**a**) Co-immunoprecipitation of COUP-TFII and THRA in nuclear proteins from skeletal muscle of WT and THRA-PV mice; COUP-TFII was used as bait. (**b**) Co-immunoprecipitation of COUP-TFII and THRA in nuclear proteins isolated from C2C12 myoblast (CTRL) and C2C12 myoblasts after COUP-TFII silencing (siCOUP-TFII); COUP-TFII was used as bait. ‘INPUT’ is the total nuclear fraction, ‘1st wash’ is the negative fraction after precipitation, ‘3rd wash’ is the last wash, ‘CoIP’ is the positive fraction after 3 washes. (**c**) ChIP- qPCR analysis of the recruitment of THRA to the TRE in the *MyoD1, Ucp3* and *Ppargc1a* promoter regions. Bar graph showed the enrichment of DNA fragments pulled down by THRA antibody. Data are shown as mean ± SEM. Two-tailed Student’s *t* test; **p < 0.01, ****p < 0.0001. Fold Change is indicated.

We utilized co-immunoprecipitation to show that COUP-TFII and THRA physically interacted in the nucleus of proliferating myoblast from THRA-PV and WT mice (Fig. 2a, S6). COUP-TFII interaction with THRA-PV appears to be stronger than with THRA (based on band size). We confirmed similar interaction in C2C12 myoblasts (Fig. 2b, S7), and after silencing COUP-TFII by siRNA this interaction was not detected (Fig. 2b, S7). The interaction between COUP-TFII and THRA was also confirmed by Proximity Ligation Assay (PLA) by Duolink staining (Fig. S8).

To investigate whether COUP-TFII/THRA interaction had an effect on the DNA-binding ability of THRA, we performed chromatin immunoprecipitation (ChIP) on WT primary myoblasts. Following COUP-TFII knock-down, recruitment of THRA on the thyroid response elements (TREs) of T3- target genes *MyoD1*, *Ucp3* and *Ppargc1a* was significantly lower (*MyoD1* *p* < 0.0001, FC = −5.8; Ucp3 *p* < 0.0001, FC = −2.8; *Ppargc1a* *p* = 0.0077, FC = −2.7, Fig. 2c). These results suggest that COUP-TFII modulates THRA binding to TREs. Taken together, these data demonstrated that a physical and functional interaction exists between COUP-TFII and THRA in murine myoblasts.

### Loss of COUP-TFII expression improves myogenic differentiation in THRA-PV myoblasts

**Figure 3:**
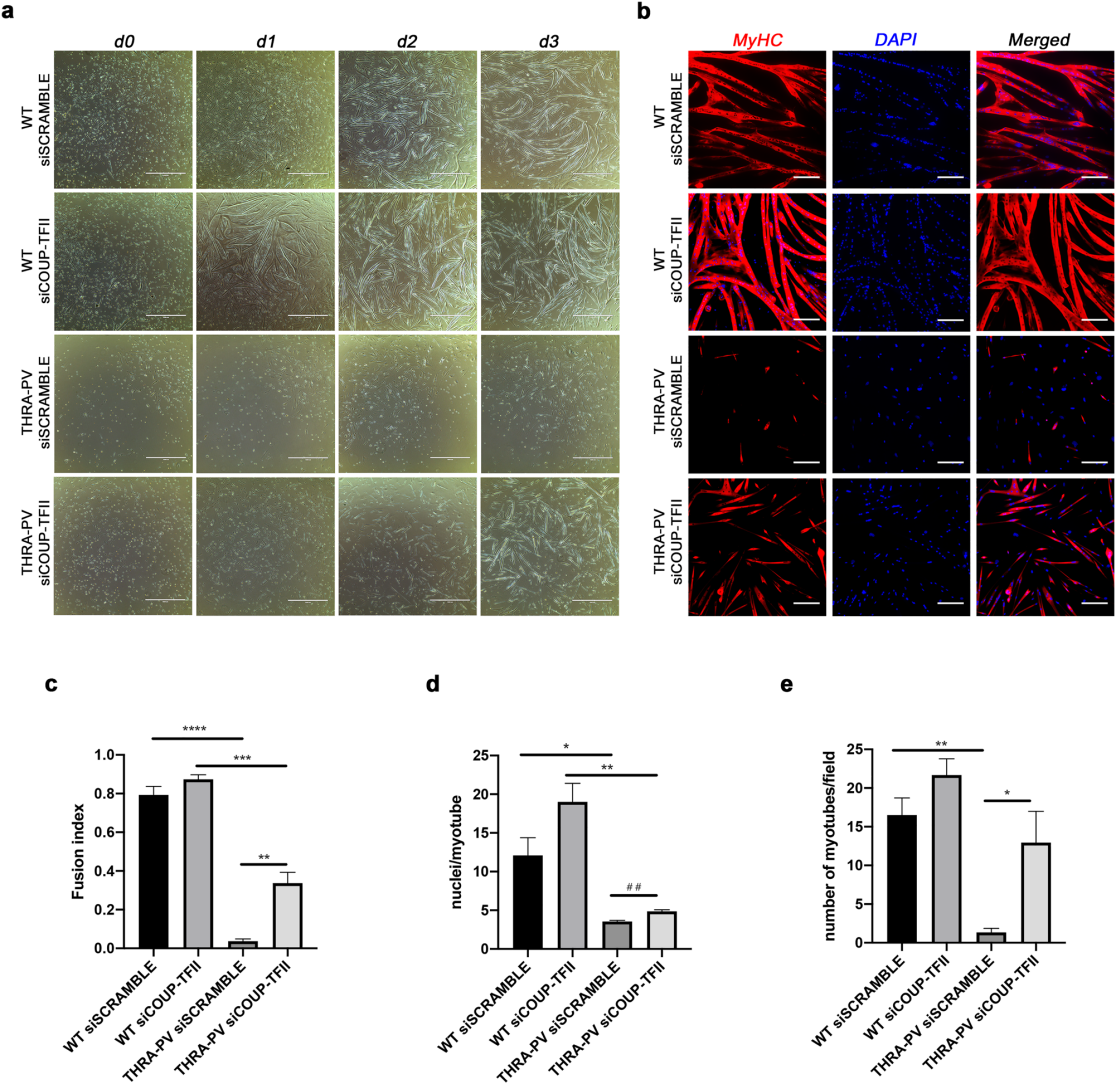
Knockdown of COUP-TFII in THRA-PV myoblasts improves myoblast differentiation. (**a**) Phase contrast images of myogenic differentiation of WT and THRA-PV myoblasts transfected with a scramble (siSCRAMBLE) siRNA or with a siRNA for COUP-TFII (siCOUP-TFII) before (d0) and after 1 (d1), 2 (d2) and 3 (d3) days of induction. Scale bar = 1000 µm (**b**) Immunostaining for Myosin heavy chain (MyHC) three days after induction of differentiation of WT and THRA-PV myoblasts transfected with a scramble (siSCRAMBLE) siRNA or an siRNA for COUP-TFII (siCOUP-TFII). MyHC in red, DAPI in blue. Scale bar = 200 µm (**c**) Fusion index, (**d**) number of nuclei per myotube and (**e**) number of myotube per field for control (siSCRAMBLE) and COUP-TFII silenced (siCOUP-TFII) in primary myoblasts from WT and THRA-PV mice three days after induction of differentiation. Data are shown as mean ± SEM, n = 3. One-way ANOVA *p < 0.05, **p < 0.01, ***p < 0.001, ****p < 0.0001. Student’s t Test ^##^* p* < 0.01.

Overexpression of COUP-TFII has been shown to repress myogenesis^14,15^, and THRA-PV mice have impaired post-natal myogenesis^7^. Here, we show that THRA-PV mice have higher expression of COUP-TFII in the skeletal muscle compared with WT. To determine if COUP-TFII mediates the defective myogenesis observed in THRA-PV-derived myoblasts, we silenced COUP-TFII protein by siRNA in primary myoblast and induced their differentiation into myotubes. The successful knockdown of COUP-TFII protein expression after siRNA silencing was possible in both WT and THRA-PV myoblasts (Supplementary Table S1). The 2 cell populations (THRA-PV and WT myoblasts) appear to be similar in term of main SCs-specific markers, such as Pax7 and MyoD, even after COUP-TFII knock-down (Tables S2 and S3). We also didn’t observe changes in the expression of genes related to innate immune response reported to be upregulated by RNA interference^16^ (Tables S4 and S5). Phase contrast microscopy imaging showed a significant impaired myogenic differentiation in THRA-PV myoblasts, as indicated by a lower myotube formation at day 3 of differentiation (Fig. 3a). The silencing of COUP-TFII in THRA-PV myoblasts resulted in improved differentiation, leading to an increase in number of mature myotubes (Fig. 3a). Conversely, the silencing of COUP-TFII in WT myoblasts appeared to accelerate the differentiation process at day 1, but on day 3 no difference was detected. We also observed a decreased myosin heavy chain (MyHC) expression in THRA-PV myoblasts (Fig. 3b). THRA-PV myoblasts presented defective myogenesis, showing few cells staining positive for MyHC per field, and few detectable myotubes that were smaller and with only 2/3 nuclei per myotube. After COUP-TFII silencing, we observed a significant increase of MyHC expression in THRA-PV myoblasts, consistent with the improvement of myotube formation. No difference in MyHC staining was found 3 days after COUP-TFII silencing in WT myoblasts (Fig. 3b). Quantitative analysis also confirmed these gross morphological observations. Myoblast fusion at day 3 of differentiation was dramatically impaired in THRA-PV myoblasts compared to WT (*p* < 0.0001, FC = −22.2), accounting for 4.5% of WT myoblasts fusion index (Fig. 3c). After silencing COUP-TFII, myoblasts fusion in THRA-PV myoblasts was significantly increased (approximately 10 folds, *p* = 0.023, FC = +9.5) in comparison to THRA-PV myoblasts treated with scramble siRNA and reached 42.6% of WT myoblasts fusion index (Fig. 3c). Similarly, myotube size (number of nuclei per myotube, Fig. 3d) and myotube number (Fig. 3e) were significantly lower in THRA-PV myoblasts compared to WT myoblasts (*p* = 0.0272, FC = −3.4 and *p* = 0.0126, FC = −12.4, respectively). Silencing of COUP-TFII in THRA-PV myoblasts resulted in a statistically significant improvement in myotube size and number, but had no effect in WT myoblasts (Fig. 1d, Fig. 3e. The number of nuclei per myotube was found non-significantly different with one-way Anova, but resulted significant with Student’s t-test, *p* = 0.0042, FC = +1.4, Fig. 3d; myotube number *p* = 0.0485, FC = +9.7, Fig. 3e).

Together, these data demonstrate that COUP-TFII plays a crucial role in the repression of myogenic differentiation in myoblasts from THRA-PV mice and that ablation of COUP-TFII improves their myogenic potential.

### COUP-TFII silencing has separate effects on the transcriptomic profile of WT and THRA-PV myoblasts

To determine the role of COUP-TFII and its inter-relationship with THRA, we performed bulk RNA-seq analysis on proliferating control and COUP-TFII-silenced myoblasts from WT and THRA-PV mice. We detected a significant downregulation of COUP-TFII expression in silenced WT and THRA-PV myoblasts, with no differences in expression between WT and THRA-PV mice (Supplementary Table S1).

**Figure 4:**
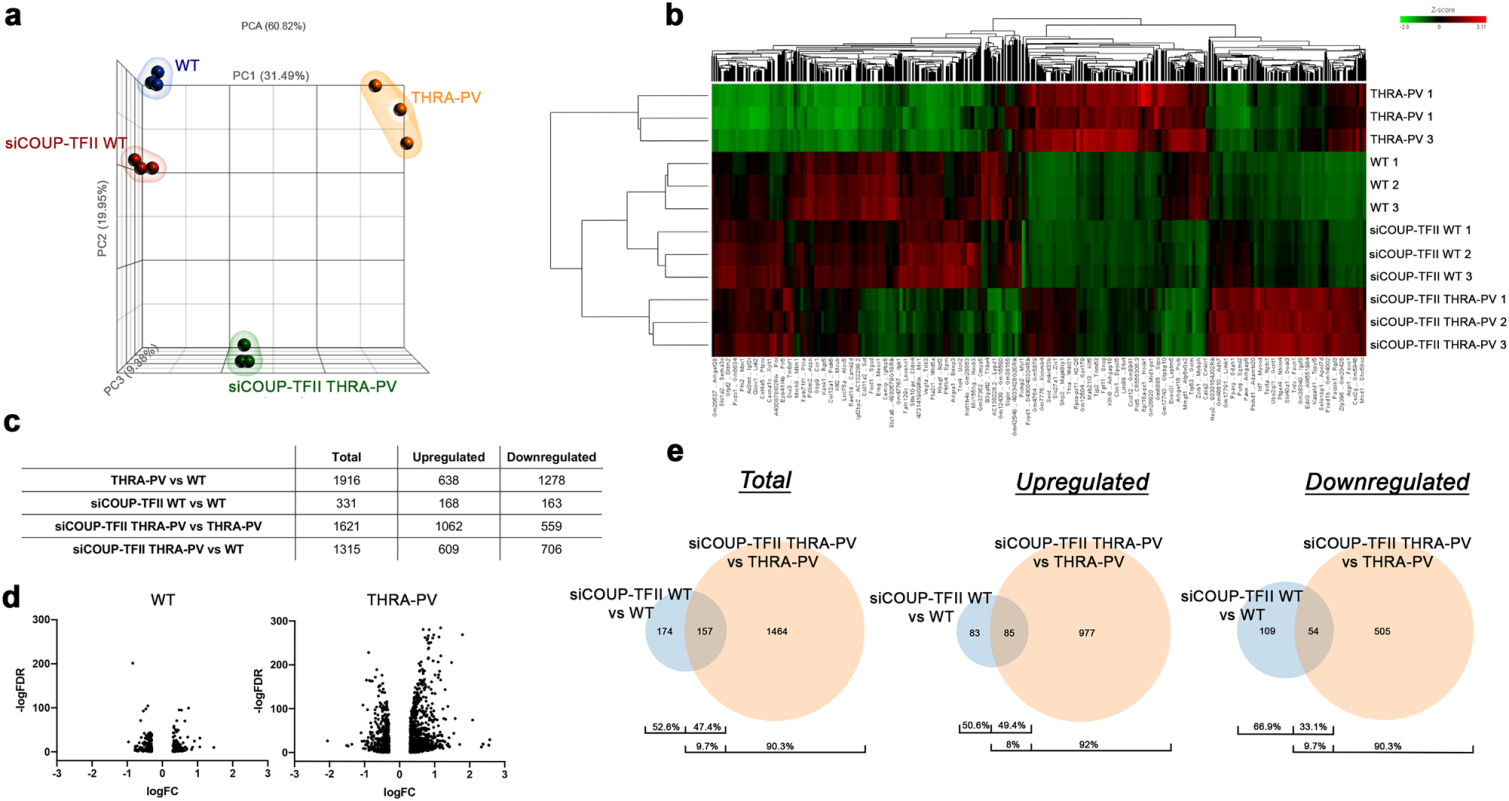
RNA-sequencing profiling of THRA-PV and WT myoblast after COUP-TFII silencing. (**a**) 3D Principal Component Analysis plot (PCA) for WT and THRA-PV myoblasts before (WT and THRA-PV) and after (siCOUP-TFII WT and siCOUP-TFII THRA-PV) COUP-TFII silencing. (**b**) Heatmap and hierarchical clustering analysis of the 3072 differentially expressed genes among WT, WT siCOUP-TFII, THRA-PV, and THRA-PV siCOUP-TFII. Downregulated genes are represented in green, upregulated genes are represented in red. (**c**) Number of total, upregulated and downregulated differentially expressed genes in THRA-PV myoblasts (THRA-PV vs WT) and after COUP-TFII silencing in WT (siCOUP-WT vs WT) or THRA-PV (siCOUP-TFII THRA-PV vs THRA-PV) myoblasts and siCOUP-TFII THRA-PV vs WT (**d**) Volcano plot representation of differential expression analysis of genes after COUP-TFII silencing in WT and THRA-PV myoblasts. The x-axis shows log_10_fold-changes in expression and the y-axis the log_10_ odds of FDR. (**e**) Venn diagrams representing summary of the total differentially expressed, upregulated and downregulated genes after COUP-TFII silencing in WT and THRA-PV myoblasts.

The Principal Component Analysis (PCA, Fig. 4a) indicated that the replicates had successful reproducibility and the different groups clustered separately. Principal Component 1, 2 and 3 (PC1, PC2, and PC3) explained 60.82% of the variance. PC1 explaining 31.49% of the variance, separates WT and WT siCOUP-TFII from THRA-PV, and PC2, explaining 19.95% of the variance, separated WT and THRA-PV from THRA-PV siCOUP-TFII. Thus, WT and THRA-PV positioned differently in the PCA sections. In addition, WT and WT siCOUP-TFII positioned together in the same section of the PCA, while THRA-PV and THRA-PV siCOUP-TFII positioned differently. Thus, indicating that a significant difference in the THRA-PV gene expression profile occurs after COUP-TFII silencing.

Hierarchical clustering analysis of differentially expressed genes (DEGs, *p* < 0.05, and |FC|> 2.0, Fig. 4b) further confirmed the observations from the PCA. WT and siCOUP-TFII WT samples clustered together, while THRA-PV and siCOUP-TFII THRA-PV diverged. THRA-PV siCOUP-TFII clusters were closer to WT and siCOUP-TFII WT than to THRA-PV.

Gene expression profiling in myoblast between WT and THRA-PV showed 1916 DEGs (Fig. 4c). After COUP-TFII silencing, THRA-PV myoblasts showed 1621 DEGs, and WT mice had 331 DEGs (Fig. 4c). A large portion of DEGs between THRA-PV and WT were downregulated, while COUP-TFII silencing in THRA-PV cells mostly induced upregulation (67% and 66%, respectively). This difference was not observed in WT myoblasts, where the number of up- and downregulated genes was similar.

The effect of COUP-TFII silencing on THRA-PV mRNA profile compared to WT is further described by the Volcano plot representation of DEGs (Fig. 4d). Silencing of COUP-TFII on THRA-PV compared to WT myoblasts resulted in an increased number of downregulated (logFC < 0) and upregulated (logFC > 0) genes.

After COUP-TFII silencing in THRA-PV myoblasts, 9.7% of the 1621 DEGs were differentially expressed also in WT, and 90.3% were differentially expressed uniquely in THRA-PV (Fig. 4e). Focusing on the upregulated and downregulated genes, we found, respectively, 85 and 54 common genes between the two groups (siCOUP-TFII WT vs WT and siCOUP-TFII THRA-PV vs PV), which represented just 8% and 9.7% of the up- and down-regulated genes in the THRA-PV.

These data demonstrate that COUP-TFII silencing has different effects on THRA-PV and WT myoblasts and that siCOUP-TFII THRA-PV myoblasts present an expression profile closer to WT than THRA-PV myoblasts.

### COUP-TFII silencing reverses the mRNA profile of THRA-PV myoblasts

**Figure 5:**
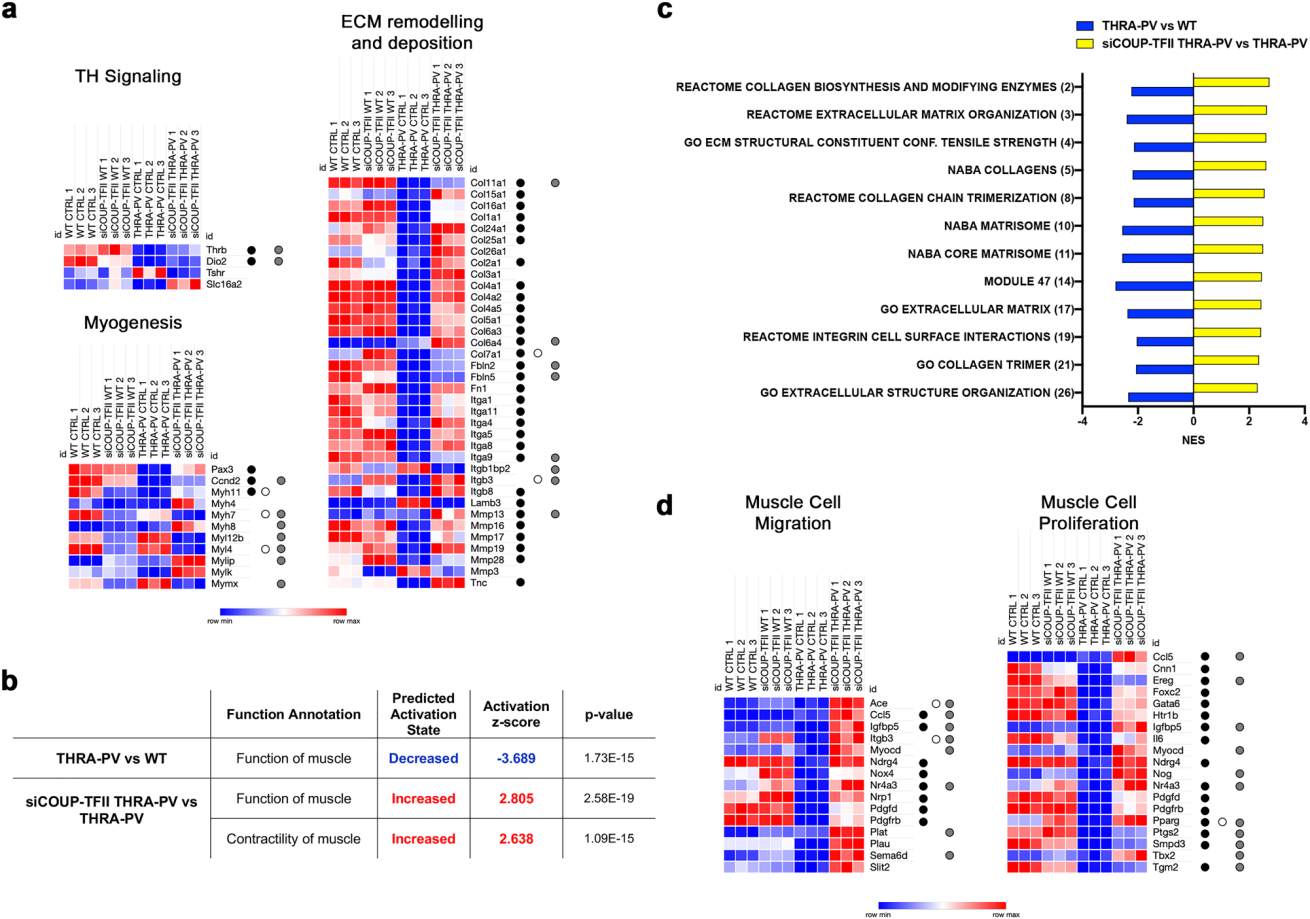
GSEA profiling of COUP-TFII silenced WT and THRA-PV myoblasts. (**a**) Heatmap representation of overall gene expression for thyroid hormone signaling, myogenic markers and ECM markers for differentially expressed genes between siCOUP-TFII THRA-PV and THRA-PV (*p* < 0.05, and |FC|> 2.0). Black dots indicate THRA-PV vs WT DEGs, white dots indicate DEGs between siCOUP-TFII WT and WT, grey dots indicate DEGs between siCOUP-TFII THRA-PV and WT. (**b**) Disease and functional analysis using the IPA knowledgebase of the THRA-PV vs WT and THRA-PV siCOUP-TFII vs THRA-PV groups focusing on the ‘Skeletal and Muscular System Development and Function’ category. Diseases and functions with z-scores > 2 or z-scores ≤ 2, and *p*-value < 0.01 were selected. (**c**) Enriched gene sets from siCOUP-TFII THRA-PV vs THRA-PV and THRA-PV vs WT comparisons. The number in brackets represents the ranking of the gene sets after GSEA of siCOUP-TFII THRA-PV vs THRA-PV. NES: normalized enrichment score. Only gene sets with FDR > 0.25 were considered. (**d**) Heatmap representation of genes related to ‘Muscle Cell Migration’ and ‘Muscle Cell Proliferation’ GO terms after leading edge analysis of DEGs of siCOUP-TFII THRA-PV cells. Black dots indicate THRA-PV vs WT DEGs, white dots indicate DEGs between WT siCOUP-TFII and WT, grey dots indicate DEGs between siCOUP-TFII THRA-PV and WT.

We first analyzed the expression of genes related to TH signaling and we found 4 genes that were differentially expressed after COUP-TFII silencing in THRA-PV myoblasts, but not in WT myoblasts (*Thrb*, *Dio2*, *Tsh* and *Slc16a2*, Fig. 5a). *Thrb* and *Dio2* were found downregulated in THRA-PV myoblasts compared to WT, but COUP-TFII silencing in THRA-PV partially reversed this effect (for TMM, FC, p value and FDR of these genes, see Supplementary Tables S6 and S7).

Then, we analyzed the expression of genes involved in myogenic differentiation. After COUP-TFII silencing in THRA-PV myoblast, a difference in gene expression of *Pax3*, cyclin D2 (*Ccdn2)*, genes coding for different myosin isoforms, myosin light chain kinase (*Mylk)* and Myomixer (*Mymx*) was detected (Fig. 5A). *Pax3*, *Ccnd2,* and *Myh11* were significantly downregulated in THRA-PV myoblasts compared to WT, and this effect was reversed after the silencing of COUP-TFII. After COUP-TFII silencing, *Pax3* and *Myh11* resulted non-significantly different from WT. Only 3 myosin genes (*Myh11*, *Myh7,* and *Myl4*) were significantly different after COUP-TFII silencing of WT myoblasts, and *Myh11* behave diametrically opposite in WT and THRA-PV after COUP-TFII silencing (Supplementary Tables S8 and S9).

Analysis of DEG after COUP-TFII silencing in THRA-PV myoblasts also identified 36 DEG involved in the extracellular matrix (ECM) remodeling and deposition. Among these genes, 30 DEG were present between THRA-PV and WT myoblasts. 28 genes were upregulated in siCOUP-TFII THRA-PV vs THRA-PV and downregulated in THRA-PV vs WT, while one showed opposite trend (*Lamb3*), and just one (*Col6a4*) was upregulated in both. Only 2 genes of this list were found to be significantly different after COUP-TFII silencing in WT myoblast, *Col7a1* and *Itgb3*, but the effect in THRA-PV myoblast resulted to be higher. In addition, the expression of 28 genes in siCOUP-TFII THRA-PV resulted non-significantly different from WT (Supplementary Tables S10 and S10).

We intersected the THRA-PV vs WT DEGs with siCOUP-TFII THRA-PV vs THRA-PV DEGs and siCOUP-TFII WT vs WT DEGs and we observed that the number of genes having an opposite trend (up- vs downregulation) after COUP-TFII silencing was different between WT and THRA-PV. These genes were 97% in the siCOUP-TFII THRA-PV, while just 18% in WT, as detailed in Supplementary Fig. S9.

We performed IPA analysis using the ‘Skeletal and Muscular System Development and Function’ category (Fig. 5b). A significant decrease in the *function of muscle* was predicted in THRA-PV compared to WT. However, after COUP-TFII silencing in THRA-PV cells, a significant increase of genes in the *function of muscle* and *contractility of muscle* were detected compared to THRA-PV myoblasts.

Next, using gene set enrichment analysis (GSEA) on DEGs, we identified the top-50 gene sets significantly enriched in siCOUP-TFII THRA-PV compared to THRA-PV. 18 gene sets showed ECM- and collagen- related genes (see FDR, NES, and ranking in Supplementary Table S11). 12 of these gene sets showed opposite normalized enrichment score (NES) when GSEA was run on the THRA-PV vs WT DEGs list (Fig. 5c). For a full list of ECM/collagen- related gene sets showing opposite effects in the two groups, see Supplementary Table S12. When we analyzed the list of gene sets significantly enriched in siCOUP-TFII THRA-PV compared to WT, we found no ECM- or collagen-related gene sets.

Leading-edge analysis of siCOUP-TFII THRA-PV identified two enriched muscle-related gene sets, ‘*Muscle Cell Migration*’ (NES = 1.8884, FDR = 0.1562) and ‘*Muscle Cell Proliferation*’ (NES = 1.7256, FDR = 0.2494). We found 15 genes involved in muscle cell migration significantly upregulated in siCOUP-TFII THRA-PV cells (Fig. 5d, Tables S10 and S11), 8 were also significantly downregulated in THRA-PV compared to WT cells, and 5 resulted non-significantly different from WT after COUP-TFII silencing. Of the 19 genes involved in muscle cell proliferation and upregulated in siCOUP-TFII THRA-PV myoblasts, 16 were significantly downregulated in THRA-PV compared to WT cells, and 6 resulted non-significantly different from WT after COUP-TFII silencing (Fig. 5d, Supplementary Table S12 and S13). Only 2 and 1 of the genes from ‘*Muscle Cell Migration*’ and ‘*Muscle Cell Proliferation*’ GO terms were also differentially expressed in WT cells after COUP-TFII silencing (Supplementary Tables S13-S16).

Therefore, these data highlight that COUP-TFII silencing in THRA-PV myoblasts reverses the mRNA profile and reactivates pathways involved in muscle function and ECM remodeling/deposition.

## Discussion

THRA is the dominant thyroid receptor isoform in skeletal muscle. THRA-PV mice (a model of RTH) carry a frame-shift mutation in the THRA gene^9^ and present a muscular phenotype consisting of small myofibers, impaired regeneration after injury^7^ and significant muscle loss with aging^8^.

We report that COUP-TFII expression in WT mice decreases during muscle maturation and aging. However, THRA-PV mice skeletal muscle presents a higher COUP-TFII expression that fails to decrease during aging compared to WT mice. Sustained COUP-TFII expression is reported to impair skeletal muscle development and hinder adult myogenesis in mice^15^. COUP-TFII expression is also found significantly higher in skeletal muscle of Duchenne Muscular Dystrophy (DMD) patients and the mouse model of DMD (mdx mice). Downregulation of COUP-TFII expression is shown to attenuate the onset of myopathic changes^15^ and is required for SCs to enter the myogenic program. Constitutive COUP-TFII expression has been shown to delay the differentiation of C2C12 myoblasts in vitro and to impair muscle regeneration after injury *in vivo*^14^. Since we detected elevated expression of COUP-TFII in the THRA-PV mice, we propose that COUP-TFII might have a role in THRA-PV skeletal muscle phenotype. The failure of COUP-TFII expression to decrease during differentiation in myoblasts derived from THRA-PV mice compared to healthy littermates suggests a possible role of COUP-TFII in halting myogenic differentiation. Silencing COUP-TFII expression during in vitro myogenic differentiation partially recovered the phenotype of THRA-PV myoblasts and significantly improved myogenesis, confirming its involvement in the THRA-PV skeletal muscle phenotype.

Transcriptomic data confirmed that COUP-TFII silencing exerts significant transcriptomic changes in THRA-PV myoblasts. In contrast, the effect of COUP-TFII silencing on the myogenic potential of WT myoblasts is negligible. COUP-TFII reduction drove THRA-PV cells transcriptome closer to WT cells, suggesting a ‘recovery’ effect. Leading-edge analysis of THRA-PV myoblasts after COUP-TFII silencing showed two enriched muscle-related gene sets, the *‘Muscle Cell Proliferation’* and ‘*Muscle Cell Migration*’. This mirrors the upregulation of genes associated with specific muscle-related and ECM-related functions. SCs involvement in muscle regeneration includes cell activation/proliferation, adhesion, migration, and anchorage-dependent growth, with ECM and integrins serving as a substratum for these functions^17^. While COUP-TFII regulation of collagen isoforms, laminins and metalloproteinases has been previously reported^18–20^, little is known on the effect of TH signaling on ECM remodeling and deposition^21,22^. SCs are known to secrete collagens type I and III. Collagen type I is mostly expressed by quiescent and proliferating myoblasts, and during differentiation this expression is decreased, highlighting the dynamic role of collagens in SC self-renewal and proliferation^23^. In myoblasts of THRA-PV compared to WT, we noted a significant decrease in gene expression of several collagens, including Collagen type I, which was recovered with COUP-TFII silencing. Integrins on cells surface promote adhesion and signaling through interaction with collagens, fibronectin, and laminin. THRA-PV myoblasts showed a significant reduction in gene expression of Integrin α1 (*Itgα1*), Integrin α4 (*Itgα4*), Integrin α5 (*Itgα5*) which was completely recovered after COUP-TFII silencing. Even if Integrin α7β1 is initially highly expressed in differentiated myotube, a switch in integrin signature is needed for proper myoblast proliferation and differentiation^24^. Itgα4 expression is induced in SC right after skeletal muscle injury to promote SCs’ activation and proliferation^25^, while Itgα5, has a crucial role in SCs’ migration and adhesion^26^.

We showed a physical interaction between COUP-TFII and THRA in myoblasts and this interaction seems to be stronger in THRA-PV mice as suggested by the Co-Ip experiment. Moreover, we demonstrated by ChIP assay that COUP-TFII influences endogenous THRA DNA binding. These data suggest that physical interaction between THRA and COUP-TFII in the nucleus have collaborative effects on modulating gene regulation. In many models, the dominant negative action of mutant THR is due to irreversible interaction with corepressor, not disrupted by ligand, and this usually translate with inhibition of thyroid hormone-mediated transcription activity^27^. Here, COUP-TFII may participate to this interaction, and the increased/aberrant recruitment of COUP-TFII by THRA-PV may work as a repressor complex. In this contest, COUP-TFII downregulation may decrease THRA-PV- negative gene regulation, inducing gene de-repression. Other factors (co-repressors and co-activators) known to cooperate with both COUP-TFII and THRA may be involved. COUP-TFII binding to DNA can result in repression on gene expression by recruitment of co-repressor such as nuclear co-repressor (NCoR)^28^ and retinoid and thyroid receptors (SMRT)^29,30^. THRA-PV also interacts with the same corepressors and forms the HDCA corepressor complexes for transcriptional repression of T3-positively-regulated genes^31,32^. Moreover, COUP-TFII can interact with other nuclear receptors and transcription factors to repress their activity, competing for the same DNA response elements or heterodimerization cofactors (RXR)^29^. It has been showed that COUP-TFII can also form heterodimers with the THRA in the context of binding to a palindromic thyroid hormone-responsive element^33^. It may be possible that COUP-TFII plays a role in this interaction.

In conclusion, we have shown that COUP-TFII and THRA cooperate during myoblast differentiation and that COUP-TFII may play an essential role in the impaired regeneration and muscular phenotype of THRA-PV mice.

## Methods

### Mice

All animal studies were performed in accordance to experimental protocols approved by the Institutional Animal Care and Use Committee (IACUC) of VA Greater Los Angeles Healthcare System and Children’s Hospital Los Angeles. The study was carried out in compliance with the ARRIVE guidelines. Animal handling was also performed according to guidelines for laboratory animal care. The study used 1 to 20-month old males and females THRA-PV, age matched wild-type (WT) siblings, and C57BL6 mice (Jackson Laboratory). The generation of the THRA-PV mouse has been previously described^9^.

### Primary myoblast isolation, cell culture and differentiation

Primary murine myoblasts were isolated using a modified previously described protocol^34^. Briefly, muscle was isolated from male and female THRA-PV and WT mice at different age. Initially, animals were euthanized by CO_2_ exposure and hind-limb muscles were removed. Muscles were cleaned, minced, and then digested twice in medium containing 0.2% Collagenase type II (Worthington, Cat# LS004176) and 0.55 U/ml Dispase (Gibco, Cat #17105-041) for 40′ at 37 °C. After digestion, tissue was passed thought a 16-gauge needle and filtered through a 100 μm and a 40 μm mesh to obtain a single-cell suspension. Cells were resuspended in DMEM/F12 with 10% horse serum (HS), 20% FBS and 0.2% Primocin (InvivoGen, Cat # ant-pm-1) and placed into non-coated 100 mm tissue-culture petri dish for 3 h to let fibroblasts attach. Cells in suspension were then transferred to laminin-511 (Laminin iMatrix-511 E8, Amsbio) coated tissue-culture petri and incubated at 37 °C and 5% CO_2_. After 48 h, medium was replaced with fresh medium. Further medium changes were performed by replacing 50% of the medium every 48 h to ensure the maintenance of “conditioning” factors for all the duration of the experiments.

C2C12 murine myoblasts (ATCC CRL-1772) were maintained in DMEM supplemented with 10% FBS. Both C2C12 cells and primary myoblasts were maintained at sub-confluent density. To induce myogenic differentiation, medium (DMEM/F12 for primary myoblasts, DMEM for C2C12) with 2% HS was added when the cells were approximately 70% confluent.

### Gene silencing

C2C12 cells and primary myoblasts were plated at a density of 1500 cells/cm^2^ in 6- or 12-well dish plates and transfected with SMART pool small interfering RNA (siRNA) targeting COUP-TFII or control scramble siRNA (25 nM, Horizon, Cat #M-063437-01-0100 and #D-001206-13-20 respectively) using DharmaFECT Transfection Reagent 1 (Horizon, Cat #T-2001-02) according to manufacturer’s instructions. Transfection medium was changed after 24 h. The transfection was repeated after 48 h. Knockdown efficiency was evaluated by PCR for the targeted mRNA.

### Immunofluorescence

Differentiated myotubes were fixed with 4% paraformaldehyde and treated with 0.1% Triton X-100. Tibialis anterior muscles (TAM) were fixed overnight and embedded in paraffin. For immunofluorescence staining the following primary antibodies were used: MyHC, Laminin, and COUP-TFII (detailed in Supplementary Materials). Detection of bound primary antibodies was carried out with appropriate secondary antibodies conjugated with Alexa Fluor 555 or 488 (ThermoFisher Scientific, detailed in Supplementary Materials). Nuclear DNA was counterstained with 4′,6-diamidino-2-phenylindole (DAPI). A Leica DM100 (Model DFC360 FX) microscope was used for imaging. To calculate fusion index, number of nuclei (DAPI positive) per myotube and number of myotube per field, nuclei were counted with ImageJ, while myotube were identified and counted based on positivity for MyHC. For each condition, 6 independent fields from 3 different samples were acquired.

### Western blot

Total or nuclear proteins were extracted from C2C12 cells, primary myoblasts, and skeletal muscle (tibialis anterior) using RIPA buffer (Millipore) or Nuclear Extraction Kit (Abcam, Cat ab113474). Proteins were quantified with BCA Protein Assay Kit (Abcam, Cat ab102536) and stored at − 80 °C until use. Protein electrophoresis was performed on 4–20% Tris–Glycine gels and transferred onto a 0.2 µm PVDF membrane (Biorad) and probed overnight at 4 °C with COUP-TFII antibody. Anti-histone H3 or anti ß-actin antibody were used to detect housekeeping genes. Anti-COUP-TFII and anti-Thyroid Hormone receptor α were used for detection of coimmunoprecipitation (CoIp) detection. HRP-conjugated secondary anti-rabbit, was used as secondary for COUP-TFII and H3 antibodies, while VeriBlot was used for CoIp detection. For a detailed list of antibodies, see Supplementary Materials. Antigens were detected using the ECL Western Blotting detection reagents (Amersham Biosciences/GE Healthcare), impressed on GE Healthcare Amersham Hyperfilm ECL films (GE Healthcare). Data from 3 independent experiments were quantified by densitometry with ImageJ software (ImageJ, NIH). All measurements were normalized against the corresponding housekeeping gene.

### Co-immunoprecipitation

Nuclear proteins were extracted from primary myoblasts and C2C12 cells with Nuclear Extraction Kit (Abcam, Cat ab113474) and then quantified with BCA Protein Assay Kit (Abcam, Cat ab102536). Extracted proteins (referred to as ‘Input’) were incubated with COUP-TFII antibody (Cell Signaling) at 4 °C for 2 h. Protein A/G PLUS-Agarose (Santa Cruz, Cat sc-2003) were then added and incubated overnight at 4 °C according to the manufacturer’s instructions. After immunoprecipitation, the precipitate bound to the agarose beads was washed three times with PBS and suspended in 40 μl of ENE2 Extraction Buffer (Nuclear Extraction Kit, Abcam) for further analysis. Ten to twenty microliters (depending on experiment) were loaded on the gel and the samples were processed by SDS–polyacrylamide gel electrophoresis and analyzed by western blot.

### Chromatin-immunoprecipitation (ChIP) analysis

ChIP was performed in proliferating WT primary myoblasts using Magna ChIP G—Chromatin Immunoprecipitation Kit (Millipore Sigma, Cat # 17-611) following manufacturer’s instructions. Briefly, cells were fixed by 1% formaldehyde and fragmented by sonication. Anti-THRA antibody (LSBio, Cat# LS-C60119, 1:100) was then used for immunoprecipitation of DNA-THRA complexes; normal anti-rabbit IgGs (Santa Cruz, Cat# 2345) were used as control. After washing and reverse-crosslinking, the precipitated DNA and input DNA were subjected to SYBR Green based qPCR using the following primers: Ucp3 (forward) 5′-ATTAGGTTTCAGGTCAGCTGGTG-3′, (reverse) 5′- CCGGGCCTCACCATTCACT-3′; *MyoD* (forward) 5′-TCTCCAGAGTGGAGTCCGAG-3′; (reverse) 5′-GCGGTAGCACTTGGCTATCT-3′; *Ppargc1a* (forward) 5′-TGCTCAAAAGAAGTTATGTGCCC-3′, (reverse) 5′-AGGGCTTTTCAAGATCTGTCTGT-3′. Specific primers were designed to span the TREs in the target genes identified in previous publications^35–37^.The enrichment of peaks was represented as percentage of input. All the results are obtained from 3 repeats and statistical significance was determined by Student’s *t* test. *P* value less than 0.05 was considered significant.

### Duolink proximity ligation in situ assay

Proximity Ligation Assay (PLA) was carried out using the Duolink In Situ kit (Sigma-Aldrich, Cat# DUO92101), following manufacturer’s instructions. Briefly, WT primary myoblasts were seeded on laminin-511-coated chamber-slides (m-Slide well, Ibidi, Cat# 80,826) and incubated at 37 °C and 5% CO_2_. After 48 h, cells were fixed in 4% paraformaldehyde for 10 min at RT, washed in PBS and permeabilized with 0.05% Triton X-100 in PBS for 10 min at RT. Unspecific binding sites were blocked by incubating cells in 5% BSA in PBS for 30′ at RT. Cells were then incubated overnight at 4 °C with primary antibodies for COUP-TFII (Abcam, Cat# ab41859) and THRA (Sigma Aldrich, Cat# SAB4502968), in diluent buffer. Detection (Ligation and Amplification) was performed according to the manufacturer's protocol. Images were acquired with a Zeiss LSM 710 Confocal Microscope (Zeiss, Germany). Protein–protein interactions appear as red dots.

### RNA sequencing and data analysis

RNA was isolated from proliferating myoblasts isolated from 10-month-old WT and THRA-PV mice with Qiagen RNeasy Micro kit (Qiagen, Cat # 74004). Each group for RNA-seq included cells from three 6-well dish plates. The RNA-seq was performed by Illumina HiSeq 3000 Sequencing System, genome reference mm10. RNA sequencing and data analysis were performed by the UCLA Technology Center for Genomics and Bioinformatics (UCLA TCGB). The Partek flow (Partek Flow software, version 7.0: 2019 Partek Inc.), edgeR^38^, Ingenuity Pathway Analysis (IPA) (QIAGEN Inc., https://www.qiagenbioinformatics.com/products/ingenuity-pathway-analysis) and GSEA (described below) were used for bioinformatics methods and data analysis. The reads were mapped to the latest UCSC transcript set using STAR-2.6.1d^39^. The Partek E/M was used to quantify reads to an annotation model. After obtaining transcript counts, the counts were normalized by TMM. PCA was applied to the transcript counts. The differential gene expressions were examined for: THRA-PV vs WT, siCOUP-TFII WT vs. WT, siCOUP-TFII THRA-PV vs THRA-PV, siCOUP-TFII THRA-PV vs siCOUP-TFII WT, and siCOUP-TFII THRA-PV, vs WT. For all results of differential gene expression analysis, the *p*-values and fold changes (FC) filtered were applied. The filter was *p* < 0.05, and |FC|> 2.0 for all differential gene expression results. Using the list of significantly differentially expressed genes, Canonical pathway analysis, Disease and Function analysis, and Networks analysis were performed by IPA.

GSEA was used for the downstream analysis of differentially expressed gene sets. The gene symbols were converted to HGNC format^40^*.* The leading-edge analysis was performed to GSEA results to determine which subsets of genes have the highest impact on the biological process^41^.

### Statistical analysis and data presentation

All experiments were performed using 3 independent repeated experiments. Data are presented as mean ± SEM. Statistical significance was calculated using a 2-tailed unpaired Student’s t test for two sample comparison and one-way ANOVA followed by Bonferroni's Multiple Comparison Test to compare more than 2 groups. All statistical analyses were performed using Prism 8 (Prism, San Jose, CA). Statistical significance is indicated in the figure legends as *p < 0.05, **p < 0.01, ***p < 0.001 or ****p < 0.0001.

## Supplementary information

Supplementary information.

## Data Availability

The datasets generated during and/or analyzed during the current study are available from the corresponding author on reasonable request.

## References

[1] Brennan MD (2006). The impact of overt and subclinical hyperthyroidism on skeletal muscle. Thyroid.

[2] Udayakumar N, Rameshkumar AC, Srinivasan AV (2005). Hoffmann syndrome: presentation in hypothyroidism. J. Postgrad. Med..

[3] Bloise FF, Cordeiro A, Ortiga-Carvalho TM (2018). Role of thyroid hormone in skeletal muscle physiology. J. Endocrinol..

[4] Izumo S, Nadal-Ginard B, Mahdavi V (1986). All members of the MHC multigene family respond to thyroid hormone in a highly tissue-specific manner. Science.

[5] Lee J-W, Kim N-H, Milanesi A (2014). Thyroid hormone signaling in muscle development, repair and metabolism. J. Endocrinol. Diabetes Obes..

[6] Brent GA (2012). Mechanisms of thyroid hormone action. J. Clin. Invest..

[7] Milanesi A (2016). Thyroid hormone receptor α plays an essential role in male skeletal muscle myoblast proliferation, differentiation, and response to injury. Endocrinology.

[8] Milanesi A (2017). Thyroid hormone receptor alpha is essential to maintain the satellite cell niche during skeletal muscle injury and sarcopenia of aging. Thyroid.

[9] Kaneshige M (2001). A targeted dominant negative mutation of the thyroid hormone alpha 1 receptor causes increased mortality, infertility, and dwarfism in mice. Proc. Natl. Acad. Sci. USA.

[10] Pereira FA, Qiu Y, Zhou G, Tsai MJ, Tsai SY (1999). The orphan nuclear receptor COUP-TFII is required for angiogenesis and heart development. Genes Dev..

[11] Xie X, Tang K, Yu C-T, Tsai SY, Tsai M-J (2013). Regulatory potential of COUP-TFs in development: stem/progenitor cells. Semin. Cell Dev. Biol..

[12] Xie X, Qin J, Lin S-H, Tsai SY, Tsai M-J (2011). Nuclear receptor chicken ovalbumin upstream promoter-transcription factor II (COUP-TFII) modulates mesenchymal cell commitment and differentiation. Proc. Natl. Acad. Sci. USA.

[13] Lee CT (2004). The nuclear orphan receptor COUP-TFII is required for limb and skeletal muscle development. Mol. Cell. Biol..

[14] Lee H-J (2017). Dysregulation of nuclear receptor COUP-TFII impairs skeletal muscle development. Sci Rep.

[15] Xie X, Tsai SY, Tsai M-J (2016). COUP-TFII regulates satellite cell function and muscular dystrophy. J. Clin. Invest..

[16] Whitehead KA, Dahlman JE, Langer RS, Anderson DG (2011). Silencing or stimulation? siRNA delivery and the immune system. Annu. Rev. Chem. Biomol. Eng..

[17] Zammit P, Beauchamp J (2001). The skeletal muscle satellite cell: stem cell or son of stem cell?. Differentiation.

[18] Li X (2013). COUP-TFII regulates human endometrial stromal genes involved in inflammation. Mol. Endocrinol..

[19] Calonge MJ, Seoane J, Massagué J (2004). Opposite Smad and chicken ovalbumin upstream promoter transcription factor inputs in the regulation of the collagen VII gene promoter by transforming growth factor-beta. J. Biol. Chem..

[20] Navab R (2004). Expression of Chicken ovalbumin upstream promoter-transcription factor ii enhances invasiveness of human lung carcinoma cells. Cancer Res..

[21] Cohen K (2014). Thyroid hormone regulates adhesion, migration and matrix metalloproteinase 9 activity via αvβ3 integrin in myeloma cells. Oncotarget.

[22] Gonçalves Trentin A, De Aguiar CBNM, Castilho Garcez R, Alvarez-Silva M (2003). Thyroid hormone modulates the extracellular matrix organization and expression in cerebellar astrocyte: effects on astrocyte adhesion: T_3_ modulates ECM organization in astrocytes. Glia.

[23] Alexakis C, Partridge T, Bou-Gharios G (2007). Implication of the satellite cell in dystrophic muscle fibrosis: a self-perpetuating mechanism of collagen overproduction. Am. J. Physiol.-Cell Physiol..

[24] Boppart MD, Mahmassani ZS (2019). Integrin signaling: linking mechanical stimulation to skeletal muscle hypertrophy. Am. J. Physiol.-Cell Physiol..

[25] Choo H-J, Canner JP, Vest KE, Thompson Z, Pavlath GK (2017). A tale of two niches: differential functions for VCAM-1 in satellite cells under basal and injured conditions. Am. J. Physiol.-Cell Physiol..

[26] Vaz R, Martins GG, Thorsteinsdóttir S, Rodrigues G (2012). Fibronectin promotes migration, alignment and fusion in an in vitro myoblast cell model. Cell Tissue Res..

[27] Fozzatti L (2013). Nuclear receptor corepressor (NCOR1) regulates in vivo actions of a mutated thyroid hormone receptor α. Proc. Natl. Acad. Sci. USA.

[28] Bailey PJ (1997). Transcriptional repression by COUP-TF II is dependent on the C-terminal domain and involves the N-CoR variant, RIP13δ1. J. Steroid Biochem. Mol. Biol..

[29] Litchfield LM, Klinge CM (2012). Multiple roles of COUP-TFII in cancer initiation and progression. J. Mol. Endocrinol..

[30] Okamura M (2009). COUP-TFII acts downstream of Wnt/β-catenin signal to silence PPARγ gene expression and repress adipogenesis. PNAS.

[31] Yoh SM, Chatterjee VK, Privalsky ML (1997). Thyroid hormone resistance syndrome manifests as an aberrant interaction between mutant T3 receptors and transcriptional corepressors. Mol. Endocrinol..

[32] Hu X, Lazar MA (1999). The CoRNR motif controls the recruitment of corepressors by nuclear hormone receptors. Nature.

[33] Berrodin TJ, Marks MS, Ozato K, Linney E, Lazar MA (1992). Heterodimerization among thyroid hormone receptor, retinoic acid receptor, retinoid X receptor, chicken ovalbumin upstream promoter transcription factor, and an endogenous liver protein. Mol. Endocrinol..

[34] Liu L, Cheung TH, Charville GW, Rando TA (2015). Isolation of skeletal muscle stem cells by fluorescence-activated cell sorting. Nat. Protoc..

[35] Muscat GE, Mynett-Johnson L, Dowhan D, Downes M, Griggs R (1994). Activation of myoD gene transcription by 3,5,3′-triiodo-L-thyronine: a direct role for the thyroid hormone and retinoid X receptors. Nucleic Acids Res..

[36] Solanes G (2005). Thyroid hormones directly activate the expression of the human and mouse uncoupling protein-3 genes through a thyroid response element in the proximal promoter region. Biochem. J..

[37] Wulf A (2008). T3-mediated expression of PGC-1alpha via a far upstream located thyroid hormone response element. Mol. Cell Endocrinol..

[38] Robinson MD, McCarthy DJ, Smyth GK (2010). edgeR: a Bioconductor package for differential expression analysis of digital gene expression data. Bioinformatics.

[39] Dobin A (2013). STAR: ultrafast universal RNA-seq aligner. Bioinformatics.

[40] Braschi B (2019). Genenames.org: the HGNC and VGNC resources in 2019. Nucleic Acids Res..

[41] Fleming DS, Miller LC (2016). Leading edge analysis of transcriptomic changes during pseudorabies virus infection. Genom. Data.

