# Supplementary material for: Persistent COUP-TFII expression underlies the myopathy and impaired muscle regeneration observed in resistance to thyroid hormone-alpha
Source: Sci Rep. 2021 Feb 25;11:4601. doi: 10.1038/s41598-021-84080-5 (PMC7907286; doi:10.1038/s41598-021-84080-5)
Supplement: Supplementary file 1 — Supplementary information. [file 41598_2021_84080_MOESM1_ESM.pdf]

Persistent COUP-TFII Expression Underlies the Myopathy and Impaired Muscle Regeneration Observed in Resistance to Thyroid Hormone-Alpha

Paola Aguiari, *PhD*[1], Yan-Yun Liu, *PhD*[1], Astgik Petrosyan, *PhD*[2], Sheue-yann Cheng, *PhD*[3], Gregory A. Brent, *MD*[1], Laura Perin, *PhD*[2] and Anna Milanesi, *MD*, *PhD*[1*].

[1]David Geffen School of Medicine at UCLA - VA Healthcare System, Los Angeles, CA, USA; [2]GOFARR Laboratory for Organ Regenerative Research and Cell Therapeutics in Urology, Children's Hospital Los Angeles, Los Angeles, CA, USA; [3]National Cancer Institute, Bethesda, MD, USA.

Paola Aguiari: *PAguiari@mednet.ucla.edu*
Yan-Yun Liu: *yyl@ucla.edu*
Astgik Petrosyan: *APetrosyan@chla.usc.edu*
Sheue-yann Cheng: *chengs@mail.nih.gov*
Gregory A. Brent: *GBrent@mednet.ucla.edu*
Laura Perin: *LPerin@chla.usc.edu*
Anna Milanesi*: *Anna Milanesi, MD, PhD.* AMilanesi@mednet.ucla.edu
**Corresponding Author*

Supplementary Data - Figures

Fig. S1
Uncropped scan of Western Blot film from Fig. 1a with size marker indications. Upper panel: shorter exposure, lower panel: longer exposure. Samples order from the left: cells at d0 (3 samples), cells at d3 (3 samples) and cells at d5 (3 samples) after differentiation induction.

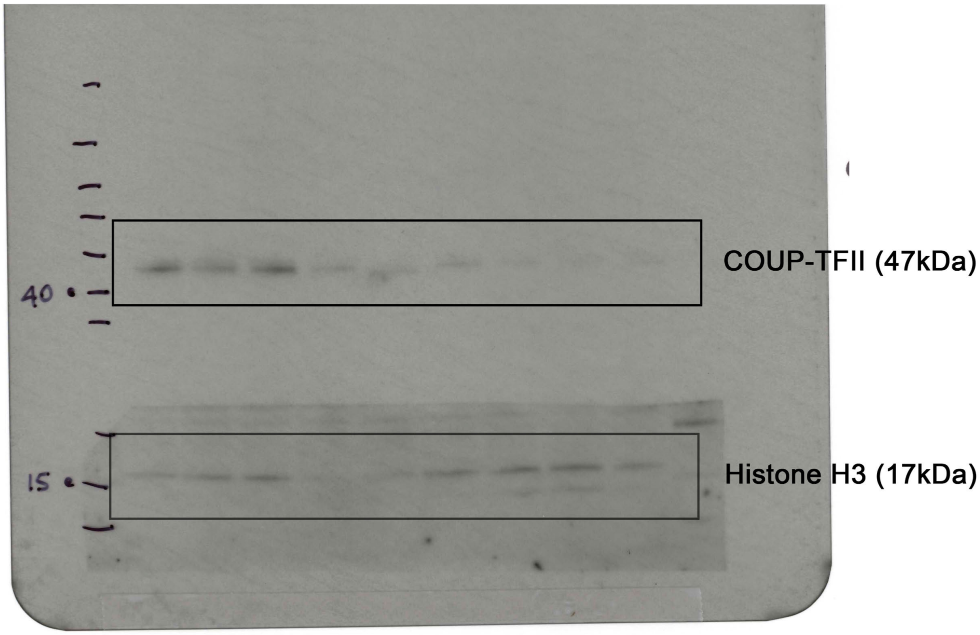

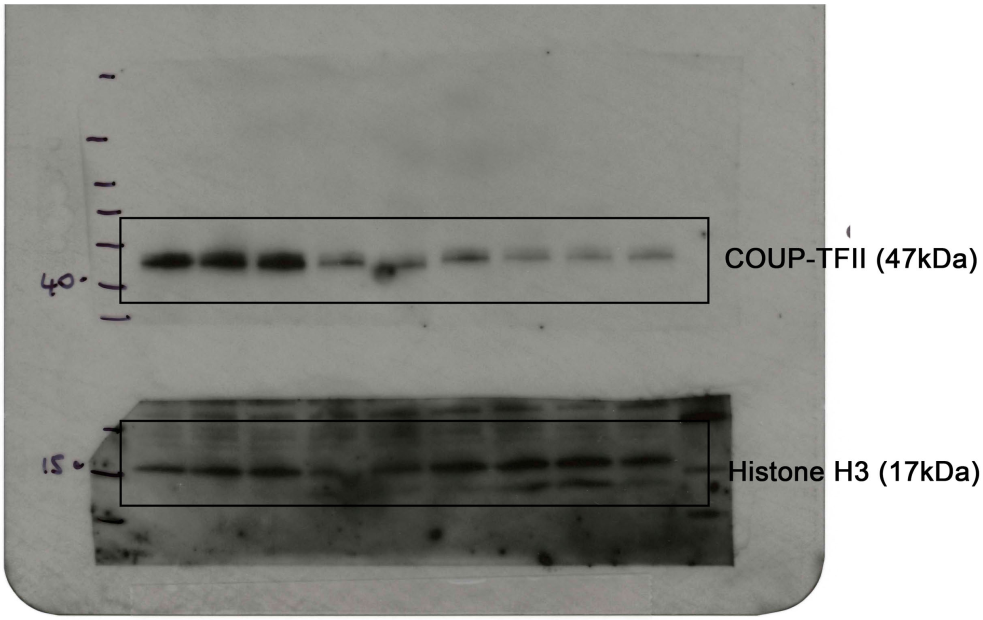

Fig. S2

Uncropped scan of Western Blot film from Fig. 1b with size marker indications. Samples order from the left (upper panel): WT skeletal muscle from 1-month-old mice (3 samples) and 2, 3 and 5-month-old mice (3 samples each, not presented in Fig 1b). Samples order from the left (lower panel): WT skeletal muscle from 10-month-old mice (3 samples) and 10-month-old mice (3 samples) [samples on the right are from an unrelated experiment].

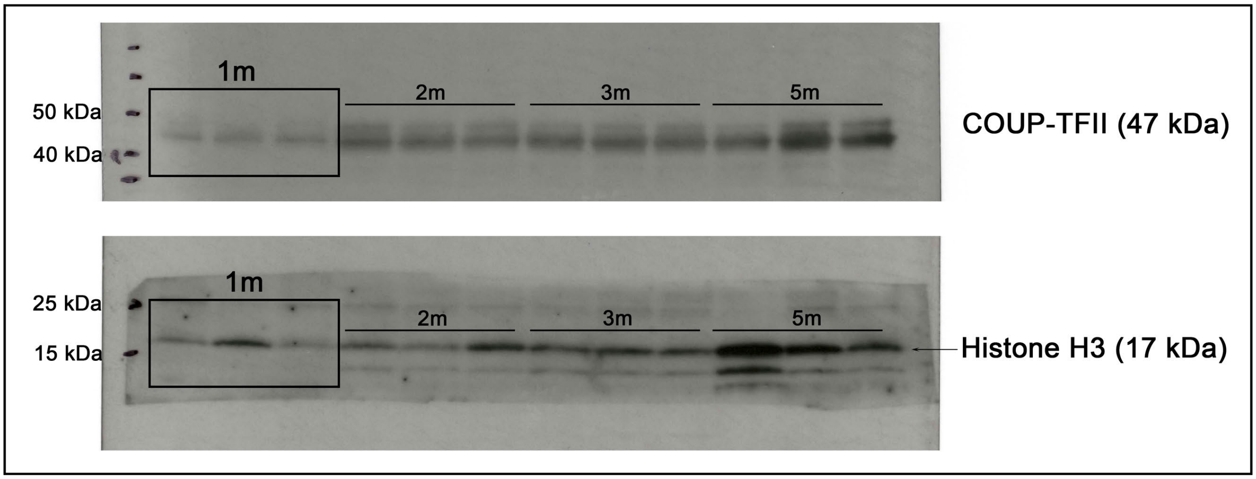

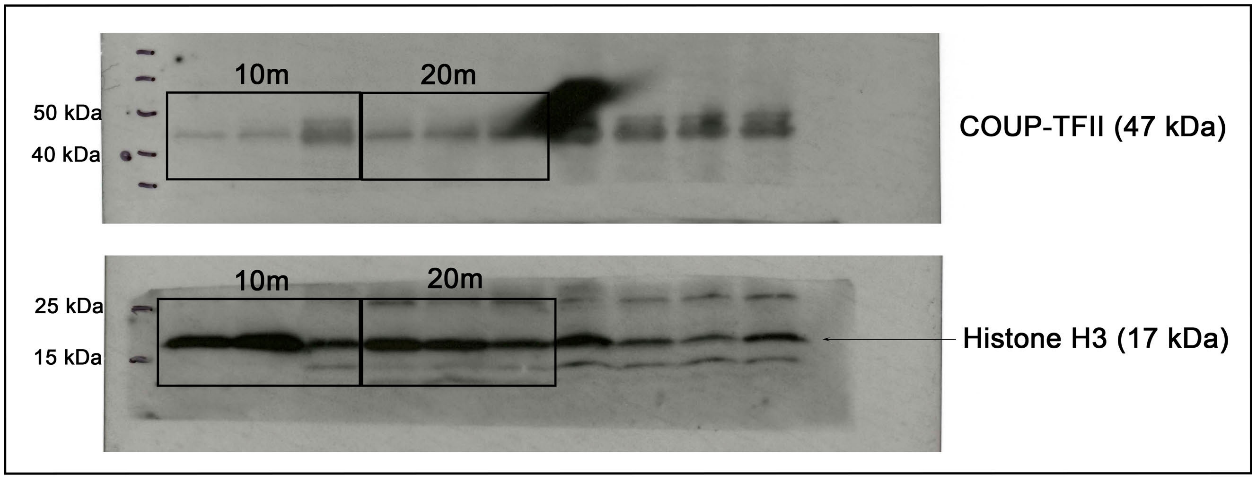

Fig. S3

Uncropped scan of Western Blot film from Fig. 1d with size marker indications. Samples order from the left: WT myoblasts at d0 (3 samples), THRA-PV myoblasts at d0 (3 samples), WT myoblasts at d5 (3 samples), THRA-PV myoblasts at d5 (3 samples) after differentiation induction.

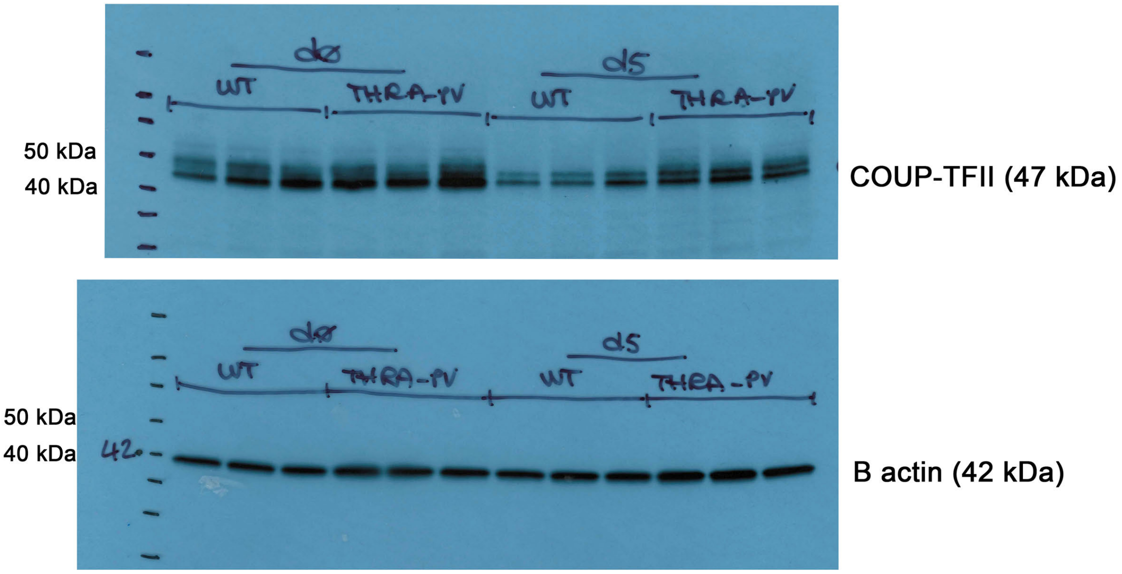

Fig. S4

Laminin (red) and nuclei (blue) immunofluorescence (A) and histogram (B) of the minimal Feret's diameter of TAM fibers from 3-months old THRA-PV mice and their WT littermates. Scale bar = 75µm. Data are shown as mean ± SEM. t Test: ****p<0.0001, FC = – 1.7.

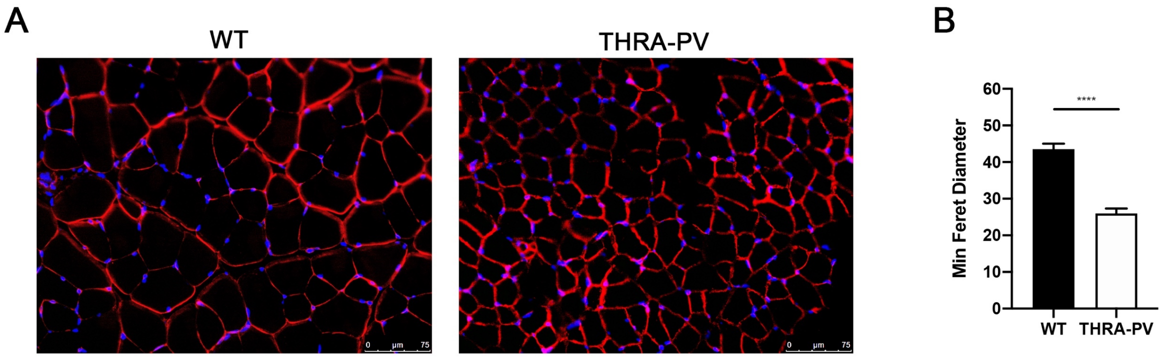

Fig. S5

Uncropped scan of Western Blot film from Fig. 1e with size marker indications. Samples order from the left (upper panel): nuclear protein extract from WT muscle from 1-month old (3 samples), 10-month old (3 samples) and 20-month old (3 samples) mice. Samples order from the left (lower panel): nuclear protein extract from THRA-PV muscle from 1-month old (3 samples), 10-month old (3 samples) and 20-month old (2 samples) mice. Samples from WT and THRA-PV mice were loaded on different polyacrylamide gels that were run and transferred together. The sample indicated with (*) was used as an internal control to normalize the bands' relative intensities between the two membranes.

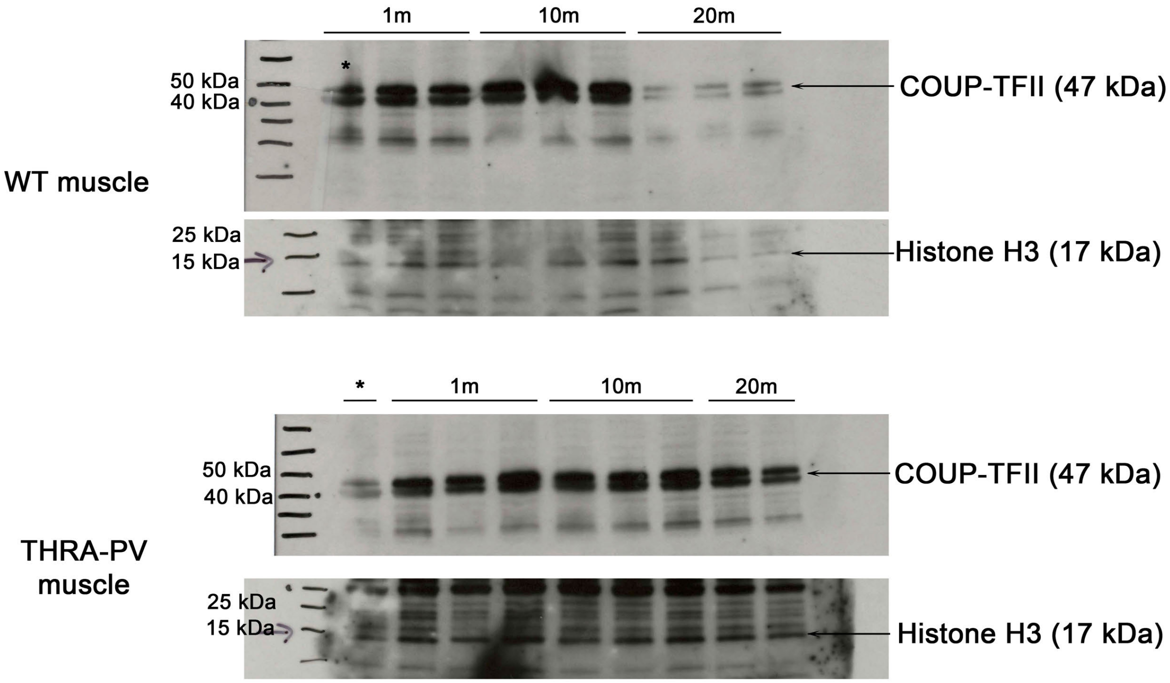

Fig. S6

Uncropped scan of Western Blot film from Fig. 2a with size marker indications. Samples order from the left: nuclear protein extract from WT myoblasts INPUT (total nuclear fraction), 1st wash (negative fraction after precipitation), 3rd wash (the last wash) and CoIP (the positive fraction after 3 washes), nuclear protein extract from THRA-PV myoblasts INPUT (total nuclear fraction), 1st wash (negative fraction after precipitation), 3rd wash (the last wash) and CoIP (the positive fraction after 3 washes). The predicted molecular weight of the band recognized by THRA antibody (Sigma-Aldrich) is 54 kDa, while the observed band size is around 48/52 kDa.

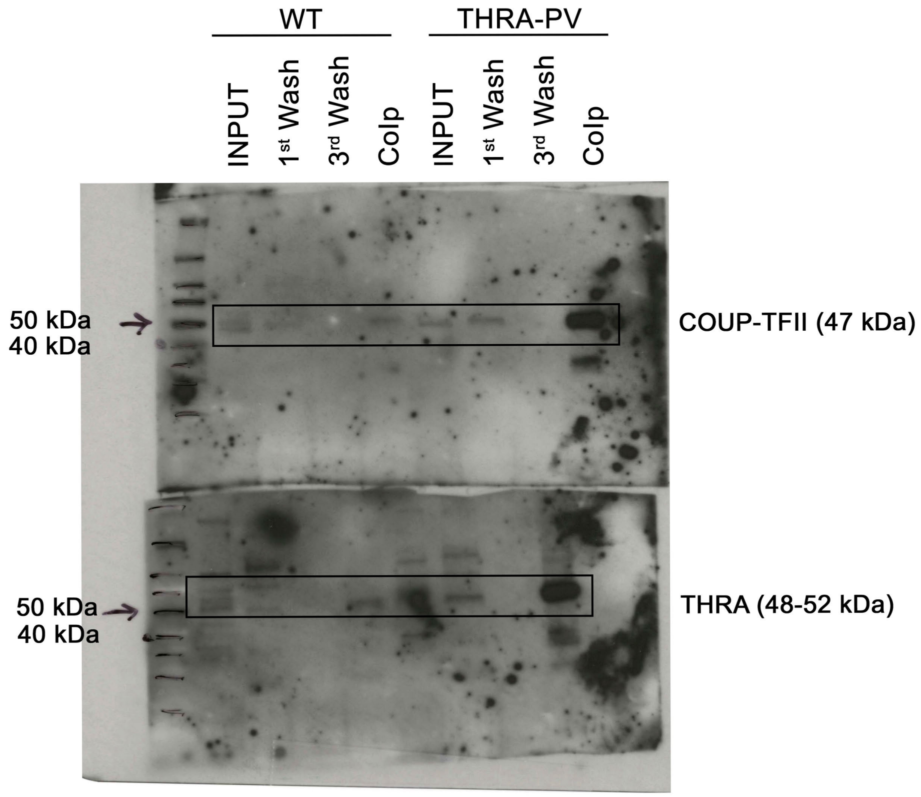

Fig. S7

Uncropped scan of Western Blot film from Fig. 2b with size marker indications. Samples order from the left: nuclear protein extract from C2C12 myoblasts: INPUT (total nuclear fraction), 1st wash (negative fraction after precipitation), 3rd wash (the last wash) and CoIP (the positive fraction after 3 washes), nuclear protein extract from C2C12 myoblasts after COUP-TFII silencing: INPUT (total nuclear fraction), 1st wash (negative fraction after precipitation), 3rd wash (the last wash) and CoIP (the positive fraction after 3 washes) [samples on the right are from an unrelated experiment]. The predicted molecular weight of the band recognized by THRA antibody (Sigma-Aldrich) is 54 kDa, while the observed band size is around 48/50 kDa.

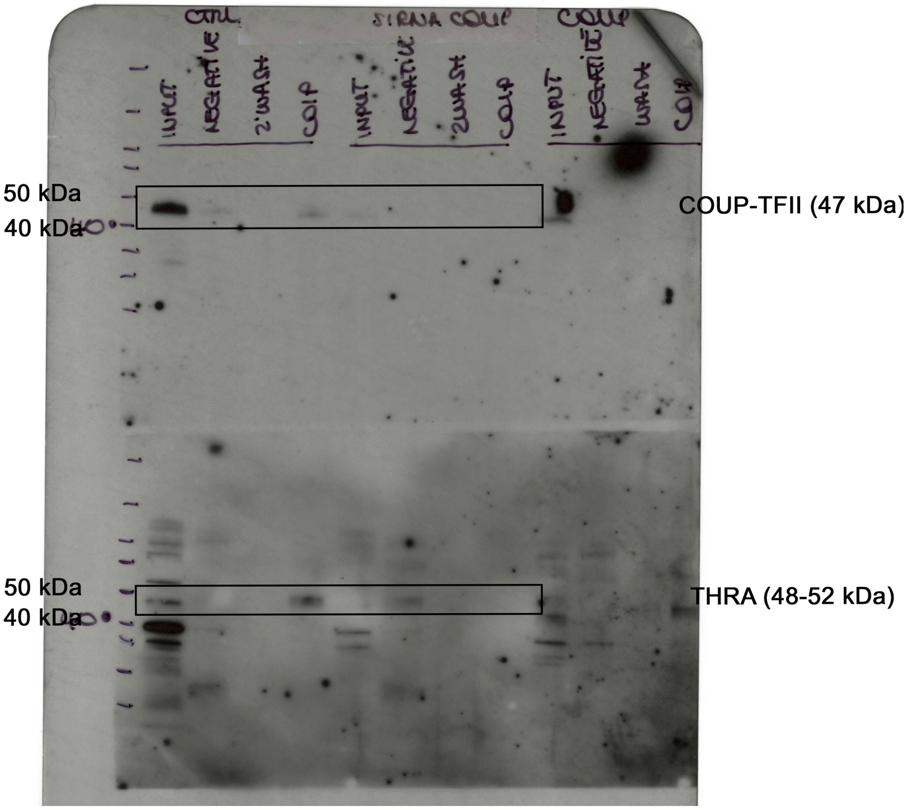

Fig. S8

Proximity Ligation Assay (PLA): red dots show COUP-TFII/THRA interaction in WT (a) and THRA-PV (d) myoblasts. (b) and (e) Confocal ortho-images of Z-stacks show that COUP-TFII and THRA interactions dots are localized within the nucleus. (c) and (f) Controls using no primary antibodies. (g) and (h) Controls using only one antibody, anti-THRA or anti-COUP-TFII. Nuclei are stained blue with DAPI. Scale-bar, 10 μm.

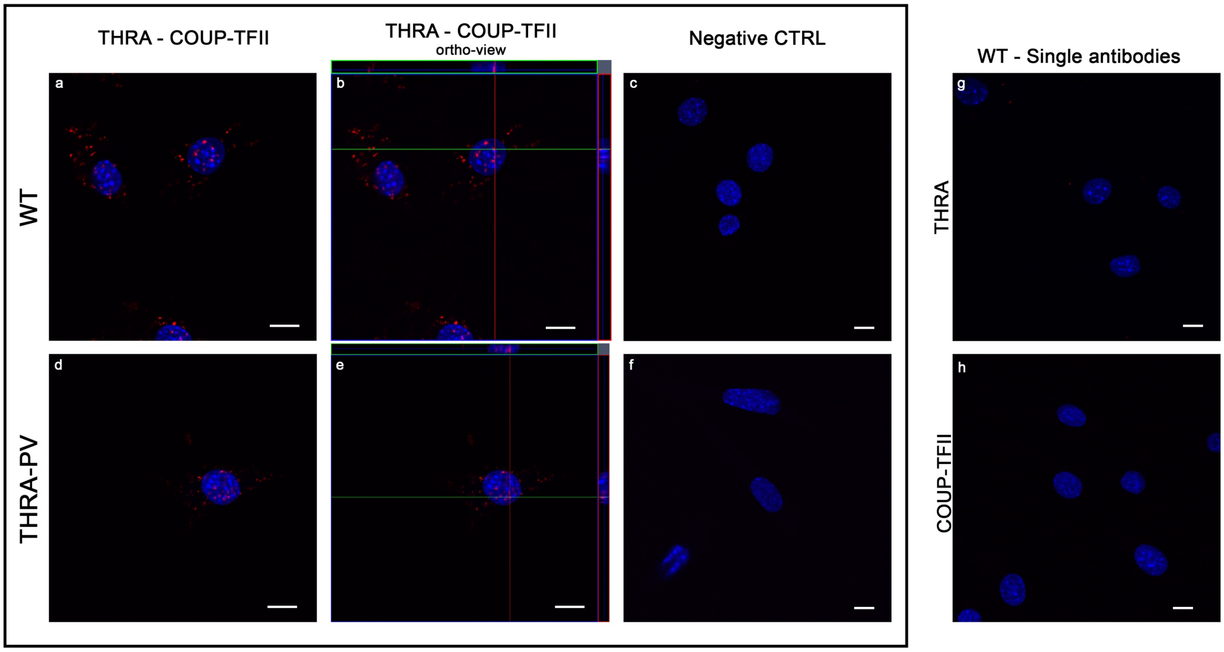

Figure S9

VENN diagrams of the DEGs, upregulated genes and downregulated genes. (A) THRA-PV vs WT DEGs intersected with siCOUP-TFII THRA-PV vs THRA-PV DEGs; of the 143 common genes, 81% (117) have the same trend in the two groups (70 upregulated and 47 downregulated in both). (B) THRA-PV vs WT DEGs intersected with siCOUP-TFII WT vs THRA-PV DEGs; of the 899 common genes, only 3% (24) have the same trend in the two groups (20 upregulated and 4 downregulated in both).

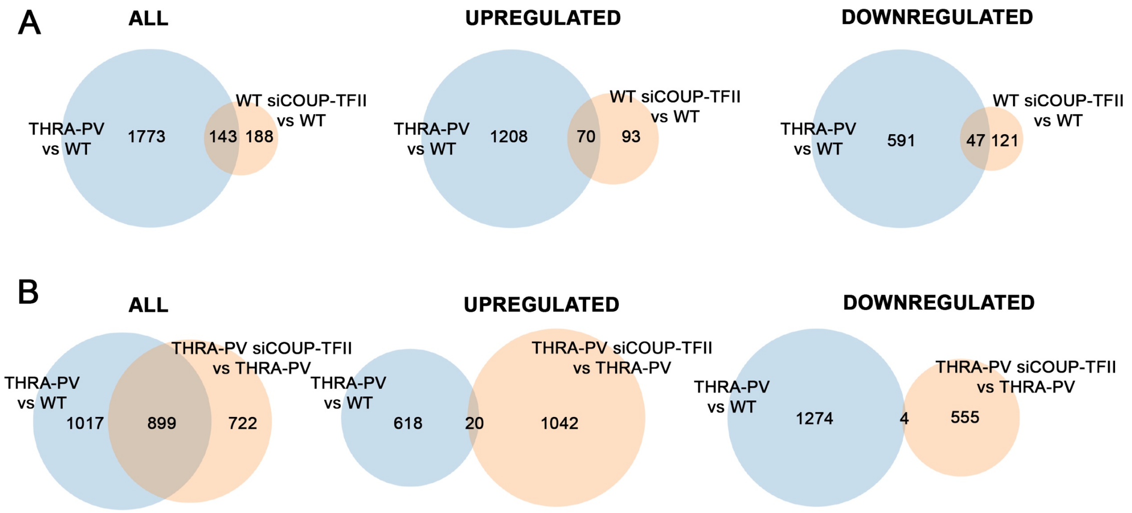

Supplementary Data - Tables

Table S1

RNAseq data for the mouse COUP-TFII gene *Nr2f2* showing the effectiveness of COUP-TFII silencing in WT and THRA-PV myoblasts. FC = fold change, FDR = false discovery rate.

	FC	p-value	FDR
**siCOUP-TFII WT vs WT**	-6.90	1.48E-206	3.85E-202
**siCOUP-TFII THRA-PV vs THRA-PV**	-7.73	2.54E-232	3.00E-229

Table S2

TMM of SCs-specific markers.

Gene	NAME	WT CTRL 1	WT CTRL 2	WT CTRL 3	siCOUP-TFII WT 1	siCOUP-TFII WT 2	siCOUP-TFII WT 3	THRA-PV CTRL 1	THRA-PV CTRL 2	THRA-PV CTRL 3	siCOUP-TFII THRA-PV 1	siCOUP-TFII THRA-PV 2	siCOUP-TFII THRA-PV 3
Pax7	paired box 7	40	46	45	50	53	54	64	73	74	121	113	122
Myod1	myogenic differentiation 1	580	572	588	576	608	616	623	583	621	970	935	982
Myf5	myogenic factor 5	13.9	13.3	13.5	8.7	9.7	10.2	14.7	14.0	12.8	19.6	21.6	20.6
Met	met proto-oncogene	94	97	104	91	92	95	151	163	169	105	96	110
Cdh15	cadherin 15	144	147	154	106	111	117	171	167	174	145	145	149

Table S3

Fold Change of SCs-specific markers.

		THRA-PV vs WT			siCOUP-TFII WT vs WT			siCOUP-TFII THRA-PV vs THRA-PV			siCOUP-TFII THRA-PV vs WT		
**Gene**	**NAME**	**FC**	**p Value**	**FDR**	**FC**	**p Value**	**FDR**	**FC**	**p Value**	**FDR**	**FC**	**p Value**	**FDR**
Pax7	paired box 7	*ns*			*ns*			*ns*			**2.7**	2.13E-91	5.67E-89
Myod1	myogenic differentiation 1	*ns*			*ns*			*ns*			*ns*		
Myf5	myogenic factor 5	*ns*			*ns*			*ns*			*ns*		
Met	met proto-oncogene	*ns*			*ns*			*ns*			*ns*		
Cdh15	cadherin 15	*ns*			*ns*			*ns*			*ns*		

Table S4

TMM for pattern recognition receptors (PRRs) and gene involved in siRNA-triggered immune response.

Gene	NAME	WT CTRL 1	WT CTRL 2	WT CTRL 3	siCOUP-TFII WT 1	siCOUP-TFII WT 2	siCOUP-TFII WT 3	THRA-PV CTRL 1	THRA-PV CTRL 2	THRA-PV CTRL 3	siCOUP-TFII THRA-PV 1	siCOUP-TFII THRA-PV 2	siCOUP-TFII THRA-PV 3
Tlr3	toll-like receptor 3	6.7	5.8	5.7	6.0	5.2	4.7	5.4	4.3	4.7	3.9	3.5	4.3
Tlr7	toll-like receptor 7	0.00	0.09	0.10	0.09	0.17	0.05	0.24	0.00	0.14	0.22	0.04	0.11
Eif2ak2	eukaryotic translation initiation factor 2-alpha kinase 2 (*PKR*)	26.3	26.1	25.2	28.3	32.6	31.2	27.1	29.9	32.9	43.2	37.9	40.9
Ddx58	DEAD (Asp-Glu-Ala-Asp) box polypeptide 58 (*RIG-I)*	23.9	24.9	25.0	26.3	26.9	27.1	33.6	36.6	35.3	28.6	30.0	28.8
Ifnar1	interferon (alpha and beta) receptor 1	72.8	71.2	67.1	81.2	81.7	80.7	81.6	79.9	89.5	97.7	92.6	95.4
Ifnar2	interferon (alpha and beta) receptor 2	31.3	32.2	30.0	34.2	36.2	37.4	25.6	26.3	28.8	33.2	30.9	30.9
Ifngr1	interferon gamma receptor 1	48.4	50.0	48.1	50.8	49.5	50.7	67.8	65.2	65.0	44.1	41.3	41.8
Ifngr2	interferon gamma receptor 2	81.2	76.0	76.4	85.9	88.8	85.3	78.7	85.5	85.3	87.6	81.2	79.8
Il12a	interleukin 12a	0.56	0.59	0.39	0.63	0.55	0.47	1.46	1.85	0.55	0.90	0.59	0.91
Il12rb1	interleukin 12 receptor, beta 1	0.65	0.68	0.29	0.99	0.77	1.04	2.12	2.41	1.45	2.40	2.85	2.56
Il12rb2	interleukin 12 receptor, beta 2	0.05	0.00	0.00	0.04	0.21	0.00	0.04	0.00	0.00	0.04	0.00	0.18
Il1a	interleukin 1 alpha	0.19	0.05	0.00	0.04	0.17	0.19	0.00	0.11	0.14	0.11	0.00	0.04
Il1b	interleukin 1 beta	1.12	1.22	0.63	0.63	0.38	0.62	1.38	0.22	0.72	0.37	0.55	0.18
Il6	interleukin 6	6.32	6.58	6.92	6.02	4.08	4.73	0.35	0.11	0.76	3.30	2.81	4.77
Tnf	tumor necrosis factor	0.00	0.09	0.10	0.18	0.47	0.24	0.51	0.82	0.24	1.57	1.00	0.91

Table S5

FC for pattern recognition receptors (PRRs) and gene involved in siRNA-triggered immune response.

		siCOUP-TFII WT vs WT			siCOUP-TFII THRA-PV vs THRA-PV		
**Gene**	**NAME**	**FC**	**p Value**	**FDR**	**FC**	**p Value**	**FDR**
Tlr3	toll-like receptor 3	0.9			0.8		
Tlr7	toll-like receptor 7	1.7			1.0		
Eif2ak2	eukaryotic translation initiation factor 2-alpha kinase 2 (*PKR*)	1.2			1.4		
Ddx58	DEAD (Asp-Glu-Ala-Asp) box	1.1			0.8		

	polypeptide 58 (*RIG-I)*						
Ifnar1	interferon (alpha and beta) receptor 1	1.1			1.1		
Ifnar2	interferon (alpha and beta) receptor 2	1.1			1.2		
Ifngr1	interferon gamma receptor 1	1.0			0.6		
Ifngr2	interferon gamma receptor 2	1.1			1.0		
Il12a	interleukin 12a	1.1			0.6		
Il12rb1	interleukin 12 receptor, beta 1	1.7			1.3		
Il12rb2	interleukin 12 receptor, beta 2	5.5	*ns*	*ns*	5.4	*ns*	*ns*
Il1a	interleukin 1 alpha	1.7			0.6		
Il1b	interleukin 1 beta	0.5			0.5		
Il6	interleukin 6	0.7			8.9	3.78E-21	7.27E-20
Tnf	tumor necrosis factor	4.7	*ns*	*ns*	2.2		

Table S6

TMM for the DEGs related to 'TH Signaling' depicted in Fig. 5a.

**Gene**	**NAME**	**WT CTRL 1**	**WT CTRL 2**	**WT CTRL 3**	**siCOUP-TFII WT 1**	**siCOUP-TFII WT 2**	**siCOUP-TFII WT 3**	**THRA-PV CTRL 1**	**THRA-PV CTRL 2**	**THRA-PV CTRL 3**	**siCOUP-TFII THRA-PV 1**	**siCOUP-TFII THRA-PV 2**	**siCOUP-TFII THRA-PV 3**
Thrb	thyroid hormone receptor beta	3.11	3.38	2.97	3.78	4.43	3.17	0.63	0.30	0.52	1.31	1.37	2.00
Dio2	deiodinase, iodothyronine, type II	28.6	31.6	29.3	17.0	18.2	18.6	2.7	1.7	3.0	8.2	5.0	7.3
Tshr	thyroid stimulating hormone receptor	0.79	1.13	1.22	0.67	1.40	0.95	2.20	1.44	2.17	0.49	0.63	0.77
Slc16a2	solute carrier family 16 (monocarboxylic acid transporters), member 2	2.56	2.35	2.58	2.97	3.70	3.22	1.93	2.15	1.93	4.61	4.07	5.19

Table S7

Fold change (FC), p value and (False Discovery Rate (FDR) for the DEGs related to 'TH Signaling' depicted in Fig. 5a.

		**THRA-PV vs WT**			**siCOUP-TFII WT vs WT**			**siCOUP-TFII THRA-PV vs THRA-PV**			**siCOUP-TFII THRA-PV vs WT**		
**Gene**	**NAME**	**FC**	**p Value**	**FDR**	**FC**	**p Value**	**FDR**	**FC**	**p Value**	**FDR**	**FC**	**p Value**	**FDR**
Thrb	thyroid hormone receptor beta	**-6.5**	2.47E-18	3.72E-17	*ns*			**3.2**	1.74E-07	1.21E-06	**-2.0**	1.24E-04	6.93E-04
Dio2	deiodinase, iodothyronine, type II	**-12.0**	1.14E-100	1.20E-98	*ns*			**2.8**	4.34E-17	6.54E-16	**-4.3**	3.75E-45	2.82E-43

Tshr	thyroid stimulating hormone receptor	*ns*			*ns*			**-3.1**	6.44E-06	3.63E-05	*ns*		
Slc16a2	solute carrier family 16 (monocarboxylic acid transporters), member 2	*ns*			*ns*			**2.3**	1.92E-09	1.64E-08	*ns*		

Table S8

TMM for the DEGs related to 'Myogenic markers' depicted in Fig. 5a.

Gene	NAME	WT CTRL 1	WT CTRL 2	WT CTRL 3	siCOUP-TFII WT 1	siCOUP-TFII WT 2	siCOUP-TFII WT 3	THRA-PV CTRL 1	THRA-PV CTRL 2	THRA-PV CTRL 3	siCOUP-TFII THRA-PV 1	siCOUP-TFII THRA-PV 2	siCOUP-TFII THRA-PV 3
Pax3	paired box 3	13.6	12.0	11.9	11.5	11.1	11.3	3.5	4.1	3.6	8.8	9.5	10.9
Ccnd2	cyclin D2	1149	1169	1108	792	796	764	131	145	153	445	419	427
Myh11	myosin, heavy polypeptide 11, smooth muscle	32.2	28.8	23.8	7.8	9.1	9.7	4.7	3.7	6.6	17.5	14.1	16.5
Myh4	myosin, heavy polypeptide 4, skeletal muscle	1.4	2.0	1.2	0.9	0.9	1.2	1.1	1.1	1.0	3.8	3.5	2.1
Myh7	myosin, heavy polypeptide 7, cardiac muscle, beta	203	214	194	64	64	72	123	128	139	43	34	23
Myh8	myosin, heavy polypeptide 8, skeletal muscle, perinatal	67	70	66	83	81	94	66	88	102	225	192	156
Myl12b	myosin, light chain 12B, regulatory	55.2	54.5	53.1	32.6	33.4	34.1	71.2	67.4	63.5	21.3	25.5	22.1
Myl4	myosin, light polypeptide 4	358	360	365	157	152	173	331	312	346	116	117	92
Mylip	myosin regulatory light chain interacting protein	9.1	8.8	8.9	17.5	15.7	15.2	12.4	12.2	13.9	26.2	28.1	27.8
Mylk	myosin, light polypeptide kinase	24.3	25.0	20.8	24.4	22.5	23.2	15.0	13.9	15.3	38.6	35.6	34.5
Mymx	NA	36.0	37.0	35.8	20.6	20.1	21.7	51.2	44.2	52.2	16.4	19.0	13.0

Table S9

Fold change (FC), p value and (False Discovery Rate (FDR) for the DEGs related to 'Myogenic markers' depicted in Fig. 5a.

		THRA-PV vs WT			siCOUP-TFII WT vs WT			siCOUP-TFII THRA-PV vs THRA-PV			siCOUP-TFII THRA-PV vs WT		
**Gene**	**NAME**	**FC**	**p Value**	**FDR**	**FC**	**FC**	**FC**	**FC**	**p Value**	**FDR**	**FC**	**p Value**	**FDR**

Pax3	paired box 3	**-3.3**	7.49E-37	2.32E-35	*ns*			**2.6**	8.51E-24	1.90E-22	*ns*		
Ccnd2	cyclin D2	**-8.0**	0	0	*ns*			**3.0**	1.33E-126	3.19E-124	**-2.13**	6.04E-53	6.07E-51
Myh11	myosin, heavy polypeptide 11, smooth muscle	**-5.6**	4.27E-51	1.94E-49	**-3.2**	9.78E-26	2.43E-23	**3.2**	3.23E-24	7.39E-23	*ns*		
Myh4	myosin, heavy polypeptide 4, skeletal muscle	*ns*			*ns*			**3.0**	3.87E-07	2.57E-06	*ns*		
Myh7	myosin, heavy polypeptide 7, cardiac muscle, beta	*ns*			**-3.0**	2.42E-34	1.12E-31	**-3.9**	6.15E-49	3.39E-47	**-6.15**	9.55E-82	2.26E-79
Myh8	myosin, heavy polypeptide 8, skeletal muscle, perinatal	*ns*			*ns*			**2.2**	1.15E-19	2.02E-18	**2.81**	1.15E-30	4.82E-29
Myl12b	myosin, light chain 12B, regulatory	*ns*			*ns*			**-2.9**	1.25E-109	2.40E-107	**-2.36**	5.52E-68	8.31E-66
Myl4	myosin, light polypeptide 4	*ns*			**-2.2**	2.97E-47	3.22E-44	**-3.0**	5.50E-86	6.48E-84	**-3.34**	1.30E-99	4.14E-97
Mylip	myosin regulatory light chain interacting protein	*ns*			*ns*			**2.1**	1.59E-33	5.45E-32	**3.07**	1.43E-61	1.89E-59
Mylk	myosin, light polypeptide kinase	*ns*			*ns*			**2.5**	1.35E-51	7.94E-50	*ns*		
Mymx	NA	*ns*			*ns*			**-3.0**	9.15E-56	6.10E-54	**-2.25**	1.49E-29	5.93E-28

Table S10

TMM for the DEGs related to ‘ECM Deposition and Remodeling’ depicted in Fig. 5a.

**Gene**	**NAME**	**WT CTRL 1**	**WT CTRL 2**	**WT CTRL 3**	**siCOUP-TFII WT 1**	**siCOUP-TFII WT 2**	**siCOUP-TFII WT 3**	**THRA-PV CTRL 1**	**THRA-PV CTRL 2**	**THRA-PV CTRL 3**	**siCOUP-TFII THRA-PV 1**	**siCOUP-TFII THRA-PV 2**	**siCOUP-TFII THRA-PV 3**
Col11a1	collagen, type XI, alpha 1	166	168	154	176	176	163	12.6	17.2	16.3	60.6	58.4	64.7
Col15a1	collagen, type XV, alpha 1	8.13	10.1	8.62	4.59	6.3	5.73	0.59	2.52	2.34	19.1	12.6	13.8
Col16a1	collagen, type XVI, alpha 1	104	98.9	95.7	130	124	123	23.8	27.1	25.9	75.2	75.2	73.6
Col1a1	collagen, type I, alpha 1	5109	5051	4548	4470	4578	4299	442	493	473	2815	2778	2863
Col24a1	collagen, type XXIV, alpha 1	2.6	3.83	3.56	5.03	4.51	3.83	0.31	0.74	1.03	5.92	5.69	5.61
Col25a1	collagen, type XXV, alpha 1	20.7	21.3	19.5	15.3	16	17.4	3.86	4.41	4.96	26.5	19.2	18.6

Col26a1	collagen, type XXVI, alpha 1	8.36	8.03	8.13	10.4	10.4	9.04	3.54	5.59	4.52	17	15.7	14.9
Col2a1	collagen, type II, alpha 1	30.6	27.2	26.6	15	15.4	18.4	7.36	11.7	6.38	28.1	23.7	22.5
Col3a1	collagen, type III, alpha 1	767	764	724	688	686	713	365	390	378	973	972	1028
Col4a1	collagen, type IV, alpha 1	3305	3285	2978	3281	3332	3298	350	420	392	2497	2558	2791
Col4a2	collagen, type IV, alpha 2	2054	2037	1845	1926	1976	1939	241	279	271	1737	1788	1910
Col4a5	collagen, type IV, alpha 5	130	136	125	143	132	129	21.9	29	24.2	96.6	96.7	111
Col5a1	collagen, type V, alpha 1	1681	1682	1548	1479	1510	1439	291	336	314	1184	1149	1128
Col6a3	collagen, type VI, alpha 3	481	475	420	496	515	478	54.3	56.9	63.6	397	345	394
Col6a4	collagen, type VI, alpha 4	1.12	1.04	1.36	1.44	1.83	1.23	3.23	3.52	4.86	10.6	9.28	9.05
Col7a1	collagen, type VII, alpha 1	62.2	67.6	68.2	166	158	143	13.4	12.1	10.2	54.5	61.6	63.9
Fbln2	fibulin 2	3051	3018	2745	2420	2482	2333	260	289	299	1101	1139	1197
Fbln5	fibulin 5	101	108	97.3	59.5	64	62.5	9.72	13.6	11.1	30.9	30.1	28.8
Fn1	fibronectin 1	7871	7935	7122	9619	10157	9690	2303	2453	2417	8273	8261	8227
Itga1	integrin alpha 1	18.9	17.1	14.8	12.2	13.6	13.7	3.58	2.67	2.31	13.3	10.1	11.3
Itga11	integrin alpha 11	15.9	17.4	15	9.93	12.8	12.9	1.3	1.04	1	12.2	8.24	10.7
Itga4	integrin alpha 4	18.1	19.7	19.5	13.1	12.4	10.9	4.33	6.11	6	21.4	17.9	17.8
Itga5	integrin alpha 5 (fibronectin receptor alpha)	933	944	869	1046	1066	1032	244	247	263	801	765	840
Itga8	integrin alpha 8	5.76	5.64	5.46	6.38	6.21	7.57	1.22	1.56	1.52	5.66	6.62	6.38
Itga9	integrin alpha 9	23.4	20.7	20.9	18.7	18.3	18.7	3.11	5.33	3.65	10.3	11.1	10.9
Itgb1bp2	integrin beta 1 binding protein 2	20.8	23.8	20.9	12.8	12.5	13.6	24.5	23.3	27.8	6.89	9.09	6.42
Itgb3	integrin beta 3	39.3	39.5	36.2	85.9	87	85.7	20.6	16.9	21.5	97.8	79.1	101
Itgb8	integrin beta 8	10.1	10.1	11.4	6.97	6.47	7.34	2.71	3.52	2.9	11.9	9.13	10.8
Lamb3	laminin, beta 3	16.2	17.2	16.4	18.5	19.4	21.2	61.1	60.1	63.4	17.3	17.2	17
Mmp13	matrix metallopeptidase 13	0.79	0.72	0.44	1.48	0.89	1.37	1.06	1.22	0.66	3.71	2.11	2.88
Mmp16	matrix metallopeptidase 16	5.9	6	4.82	4.5	5.11	5.96	1.18	1.56	1.59	4.27	4.84	4.14
Mmp17	matrix metallopeptidase 17	9.8	9.5	9.6	7.2	7.7	6.1	2.1	1.3	1.3	5.5	7.1	4.7
Mmp19	matrix metallopeptidase 19	16.1	16.2	16.4	21.2	19.2	19.2	5.9	7.04	5.86	23.5	21	21.1
Mmp28	matrix metallopeptidase 28 (epilysin)	3.5	3.6	3.0	6.3	6.5	6.0	0.6	1.1	0.2	2.0	3.0	2.9
Mmp3	matrix metallopeptidase 3	1.07	0.9	0.93	0.13	0.09	0.19	1.69	1.19	1.48	0.67	0.33	0.49
Tnc	tenascin C	2075	2082	1920	2044	2091	2073	790	819	812	3155	3043	3243

Table S11

Fold change (FC), p value and (False Discovery Rate (FDR) for the DEGs related to 'ECM Deposition and Remodeling' depicted in Fig. 5A.

		**THRA-PV vs WT**			**siCOUP-TFII WT vs WT**			**siCOUP-TFII THRA-PV vs THRA-PV**			**siCOUP-TFII THRA-PV vs WT**		
**Gene**	**NAME**	**FC**	**p Value**	**FDR**	**FC**	**p Value**	**FDR**	**FC**	**p Value**	**FDR**	**FC**	**p Value**	**FDR**
Col11a1	collagen, type XI, alpha 1	**-10.6**	0	0	*ns*			**4.0**	2.01E-127	4.90E-125	**-2.66**	1.34E-80	3.05E-78
Col15a1	collagen, type XV, alpha 1	**-4.9**	2.27E-15	2.86E-14	*ns*			**8.3**	9.61E-26	2.34E-24	*ns*		
Col16a1	collagen, type XVI, alpha 1	**-3.9**	4.20E-211	1.24E-208	*ns*			**2.9**	4.99E-132	1.31E-129	*ns*		
Col1a1	collagen, type I, alpha 1	**-10.5**	0	0	*ns*			**6.0**	1.57E-261	2.56E-258	*ns*		
Col24a1	collagen, type XXIV, alpha 1	**-4.7**	8.50E-16	1.10E-14	*ns*			**8.1**	5.30E-30	1.59E-28	*ns*		
Col25a1	collagen, type XXV, alpha 1	**-4.6**	6.16E-54	2.95E-52	*ns*			**4.8**	5.57E-58	3.97E-56	*ns*		
Col26a1	collagen, type XXVI, alpha 1	*ns*			*ns*			**3.5**	8.48E-38	3.38E-36	*ns*		
Col2a1	collagen, type II, alpha 1	**-3.3**	5.78E-27	1.32E-25	*ns*			**2.9**	4.90E-22	9.89E-21	*ns*		
Col3a1	collagen, type III, alpha 1	*ns*			*ns*			**2.6**	3.49E-121	7.96E-119	*ns*		
Col4a1	collagen, type IV, alpha 1	**-8.2**	1.10E-288	7.93E-286	*ns*			**6.7**	6.05E-243	7.88E-240	*ns*		
Col4a2	collagen, type IV, alpha 2	**-7.5**	5.13E-308	3.93E-305	*ns*			**6.9**	8.23E-285	2.68E-281	*ns*		
Col4a5	collagen, type IV, alpha 5	**-5.2**	2.48E-168	5.56E-166	*ns*			**4.1**	5.60E-124	1.30E-121	*ns*		
Col5a1	collagen, type V, alpha 1	**-5.22**	3.55E-250	1.75E-247	*ns*			**3.7**	1.93E-161	8.40E-159	*ns*		
Col6a3	collagen, type VI, alpha 3	**-7.87**	6.35E-287	4.47E-284	*ns*			**6.5**	5.78E-241	7.16E-238	*ns*		
Col6a4	collagen, type VI, alpha 4	**3.31**	1.41E-12	1.46E-11	*ns*			**2.5**	2.05E-11	2.08E-10	**8.2**	6.15E-39	3.58E-37
Col7a1	collagen, type VII, alpha 1	**-5.6**	1.04E-162	2.21E-160	**2.36**	2.68E-54	4.65E-51	**5.1**	4.58E-147	1.45E-144	*ns*		
Fbln2	fibulin 2	**-10.4**	0	0	*ns*			**4.0**	1.18E-160	4.86E-158	**-2.6**	9.77E-78	1.99E-75
Fbln5	fibulin 5	**-8.9**	3.08E-317	2.59E-314	*ns*			**2.6**	5.65E-59	4.11E-57	**-3.4**	2.52E-124	1.19E-121
Fn1	fibronectin 1	**-3.2**	5.05E-120	6.75E-118	*ns*			**3.4**	2.78E-135	7.47E-133	*ns*		
Itga1	integrin alpha 1	**-6.0**	4.12E-58	2.17E-56	*ns*			**4.1**	2.91E-36	1.10E-34	*ns*		
Itga11	integrin alpha 11	**-14.5**	1.18E-87	1.00E-85	*ns*			**9.3**	1.17E-60	8.84E-59	*ns*		
Itga4	integrin alpha 4	**-3.5**	1.41E-43	5.32E-42	*ns*			**3.5**	3.32E-44	1.62E-42	*ns*		
Itga5	integrin alpha 5 (fibronectin	**-3.7**	1.69E-182	3.99E-180	*ns*			**3.2**	4.19E-149	1.42E-146	*ns*		

	receptor alpha)												
Itga8	integrin alpha 8	**-4.0**	8.01E-25	1.65E-23	*ns*			**4.3**	4.51E-30	1.36E-28	*ns*		
Itga9	integrin alpha 9	**-5.4**	1.03E-73	7.23E-72	*ns*			**2.7**	4.54E-25	1.08E-23	**-2.0**	3.71E-17	7.83E-16
Itgb1 bp2	integrin beta 1 binding protein 2	*ns*			*ns*			**-3.4**	1.65E-49	9.22E-48	**-2.9**	7.91E-38	4.38E-36
Itgb3	integrin beta 3	*ns*			**2.25**	1.10E-39	7.75E-37	**4.7**	1.90E-130	4.90E-128	**2.4**	3.80E-47	3.06E-45
Itgb8	integrin beta 8	*ns*			*ns*			**3.5**	7.77E-31	2.44E-29	*ns*		
Lamb 3	laminin, beta 3	**3.7**	1.57E-152	2.95E-150	*ns*			**-3.6**	4.17E-156	1.58E-153	*ns*		
Mmp 13	matrix metallopeptidase 13	*ns*			*ns*			**3.0**	3.28E-06	1.93E-05	**4.4**	3.45E-09	3.68E-08
Mmp 16	matrix metallopeptidase 16	**-3.9**	1.61E-20	2.73E-19	*ns*			**3.0**	1.73E-14	2.21E-13	*ns*		
Mmp 17	matrix metallopeptidase 17	**-6.1**	7.67E-37	2.37E-35	*ns*			**3.7**	2.68E-19	4.63E-18	*ns*		
Mmp 19	matrix metallopeptidase 19	**-2.6**	2.95E-37	9.30E-36	*ns*			**3.5**	2.14E-68	1.88E-66	*ns*		
Mmp 28	matrix metallopeptidase 28 (epilysin)	**-5.3**	7.09E-15	8.59E-14	*ns*			**4.1**	4.09E-11	4.04E-10	*ns*		
Mmp 3	matrix metallopeptidase 3	*ns*			*ns*			**-2.9**	5.22E-05	0.000256708	*ns*		
Tnc	tenascin C	**-2.5**	6.68E-97	6.57E-95	*ns*			**3.9**	1.85E-202	1.50E-199	*ns*		

Table S12

ECM-related gene sets among the top-50 most enriched in siCOUP-TFII THRA-PV compared to THRA-PV (GSEA). Their rank, false discovery rate (FDR) and normalized enrichment score (NES) are listed. *Module 47: ECM and collagens.

**Rank**	**Gene set**	**FDR**	**NES**
2	REACTOME COLLAGEN BIOSYNTHESIS AND MODIFYING ENZYMES	0.001	**2.7287**
3	REACTOME EXTRACELLULAR MATRIX ORGANIZATION	0.0027	**2.6372**
4	GO EXTRACELLULAR MATRIX STRUCTURAL CONSTITUENT CONFERRING TENSILE STRENGTH	0.0023	**2.6163**
5	NABA COLLAGENS	0.0019	**2.615**
8	REACTOME COLLAGEN CHAIN TRIMERIZATION	0.0027	**2.5539**
10	NABA MATRISOME	0.0048	**2.505**

11	NABA CORE MATRISOME	0.0045	**2.5012**
12	REACTOME COLLAGEN DEGRADATION	0.0063	**2.4712**
14	MODULE 47*	0.0067	**2.4559**
17	GO EXTRACELLULAR MATRIX	0.0072	**2.4389**
19	REACTOME INTEGRIN CELL SURFACE INTERACTIONS	0.0074	**2.429**
21	GO COLLAGEN TRIMER	0.0159	**2.3541**
26	GO EXTRACELLULAR STRUCTURE ORGANIZATION	0.0233	**2.304**
29	REACTOME DEGRADATION OF THE EXTRACELLULAR MATRIX	0.0243	**2.2898**
34	REACTOME ECM PROTEOGLYCANS	0.0278	**2.2657**
35	REACTOME COLLAGEN FORMATION	0.0287	**2.26**
38	PID INTEGRIN1 PATHWAY	0.0306	**2.2417**
48	GO EXTRACELLULAR MATRIX STRUCTURAL CONSTITUENT	0.0401	**2.1962**

Table S13
ECM-related gene sets from GSEA and their normalized enrichment score (NES) for THRA-PV vs WT and THRA-PV siCOUP-TFII vs THRA-PV. *Module 47: ECM and collagens; **Module 122: Adhesion molecules. Only gene list with FDR > 0.25 were considered.

Gene set	siCOUP-TFII THRA-PV vs THRA-PV (NES)	THRA-PV vs WT (NES)
REACTOME COLLAGEN BIOSYNTHESIS AND MODIFYING ENZYMES	**2.7287**	**-2.238**
REACTOME EXTRACELLULAR MATRIX ORGANIZATION	**2.6372**	**-2.404**
GO EXTRACELLULAR MATRIX STRUCTURAL CONSTITUENT CONFERRING TENSILE STRENGTH	**2.6163**	**-2.1393**
NABA COLLAGENS	**2.615**	**-2.1899**
REACTOME COLLAGEN CHAIN TRIMERIZATION	**2.5539**	**-2.1519**
NABA MATRISOME	**2.505**	**-2.5638**
NABA CORE MATRISOME	**2.5012**	**-2.5627**
MODULE 47*	**2.4559**	**-2.8063**
GO EXTRACELLULAR MATRIX	**2.4389**	**-2.3773**
REACTOME INTEGRIN CELL SURFACE INTERACTIONS	**2.429**	**-2.0437**
GO COLLAGEN TRIMER	**2.3541**	**-2.0664**
GO EXTRACELLULAR STRUCTURE ORGANIZATION	**2.304**	**-2.3522**
REACTOME ECM PROTEOGLYCANS	**2.2657**	**-1.9388**
REACTOME COLLAGEN FORMATION	**2.26**	**-2.196**
GO EXTRACELLULAR MATRIX STRUCTURAL CONSTITUENT	**2.1962**	**-2.04**

REACTOME ASSEMBLY OF COLLAGEN FIBRILS AND OTHER MULTIMERIC STRUCTURES	**2.1713**	**-2.0783**
KEGG ECM RECEPTOR INTERACTION	**2.1335**	**-2.2238**
GO COLLAGEN CONTAINING EXTRACELLULAR MATRIX	**2.0711**	**-2.2364**
MODULE122**	**2.0108**	**-2.2292**
KEGG FOCAL ADHESION	**1.8783**	**-2.087**
NABA MATRISOME ASSOCIATED	**1.8441**	**-2.0763**
GO CELL MATRIX ADHESION	**1.8353**	**-1.8626**

Table S14

TMM for the DEGs related to *Muscle Cell Migration* depicted in Fig. 5d.

**Gene**	**NAME**	**WT CTRL 1**	**WT CTRL 2**	**WT CTRL 3**	**siCOUP-TFII WT 1**	**siCOUP-TFII WT 2**	**siCOUP-TFII WT 3**	**THRA-PV CTRL 1**	**THRA-PV CTRL 2**	**THRA-PV CTRL 3**	**siCOUP-TFII THRA-PV 1**	**siCOUP-TFII THRA-PV 2**	**siCOUP-TFII THRA-PV 3**
Ace	angiotensin I converting enzyme (peptidyl-dipeptidase A) 1	1.25	1.13	1.66	3.19	3.19	3.17	0.55	1.67	0.97	10.23	10.87	9.92
Ccl5	chemokine (C-C motif) ligand 5	0.00	0.32	0.15	0.72	0.55	0.66	3.62	1.78	2.93	18.35	20.22	14.69
Igfbp5	insulin-like growth factor binding protein 5	225	218	230	376	403	416	89	101	107	1453	1213	1674
Itgb3	integrin beta 3	39.3	39.5	36.2	85.9	87.0	85.7	20.6	16.9	21.5	97.8	79.1	101.1
Myocd	myocardin	1.12	0.90	1.07	1.17	0.98	1.51	0.90	0.48	0.52	3.18	2.66	2.28
Ndrg4	N-myc downstream regulated gene 4	61.1	61.1	57.2	58.3	58.8	56.4	19.1	22.9	24.1	61.1	55.4	58.4
Nox4	NADPH oxidase 4	13.1	14.2	13.6	26.0	24.0	23.4	3.1	3.6	5.3	14.5	15.0	15.9
Nrp1	neuropilin 1	2.79	2.62	3.41	4.50	5.91	5.30	1.30	0.37	0.62	5.06	7.47	7.61
Nr4a3	nuclear receptor subfamily 4, group A, member 3	195	193	178	265	273	276	62	65	67	231	202	228
Plat	plasminogen activator, tissue	20.8	23.8	22.5	21.8	19.0	21.2	1.3	1.8	1.4	11.2	13.0	14.5
Plau	plasminogen activator, urokinase	3323	334	303	321	332	315	59	59	59	230	193	228
Pdgfrb	platelet derived growth factor receptor, beta polypeptide	14.3	14.3	12.6	24.5	23.2	21.1	23.4	20.7	19.9	56.4	53.6	54.7
Pdgfd	platelet-derived growth factor, D polypeptide	190	190	190	189	190	197	110	105	108	262	287	292
Sema6d	sema domain, transmembrane domain (TM), and cytoplasmic domain, (semaphorin) 6D	27.6	30.2	30.6	41.2	42.1	41.0	25.2	25.9	24.5	71.4	62.8	72.3

Slit2	slit homolog 2 (Drosophila)	42.4	48.6	44.3	42.9	39.1	39.8	24.5	23.6	30.2	73.4	81.3	64.4

Table S15

Fold change (FC), p value and (False Discovery Rate (FDR) for the DEGs related to *Muscle Cell Migration* depicted in Fig. 5d.

		**THRA-PV vs WT**			**siCOUP-TFII WT vs WT**			**siCOUP-TFII THRA-PV vs THRA-PV**			**siCOUP-TFII THRA-PV vs WT**		
**Gene**	**NAME**	**FC**	**p Value**	**FDR**	**FC**	**p Value**	**FDR**	**FC**	**p Value**	**FDR**	**FC**	**p Value**	**FDR**
Ace	angiotensin I converting enzyme (peptidyl-dipeptidase A) 1	*ns*			**2.4**	1.50E-06	4.29E-05	**9.7**	1.76E-41	7.87E-40	**7.7**	6.82E-34	3.30E-32
Ccl5	chemokine (C-C motif) ligand 5	**17.3**	4.22E-26	9.13E-25	*ns*			**6.4**	6.51E-31	2.05E-29	**110.5**	7.36E-85	1.83E-82
Igfbp5	insulin-like growth factor binding protein 5	**-2.3**	7.87E-34	2.24E-32	*ns*			**14.6**	2.26E-289	9.80E-286	**6.4**	1.04E-156	9.99E-154
Itgb3	integrin beta 3	*ns*			**2.2**	1.10E-39	7.75E-37	**4.7**	1.90E-130	4.90E-128	**2.4**	3.80E-47	3.06E-45
Myocd	myocardin	*ns*			*ns*			**4.3**	5.72E-11	5.58E-10	**2.6**	6.74E-06	4.68E-05
Ndrg4	N-myc downstream regulated gene 4	**-2.7**	2.83E-84	2.34E-82	*ns*			**2.6**	8.89E-82	9.97E-80	*ns*		
Nox4	NADPH oxidase 4	**-3.4**	2.20E-38	7.14E-37	*ns*			**3.7**	6.94E-47	3.60E-45	*ns*		
Nrp1	neuropilin 1	**-2.9**	6.42E-120	8.44E-118	*ns*			**3.4**	2.82E-157	1.10E-154	*ns*		
Nr4a3	nuclear receptor subfamily 4, group A, member 3	**-3.9**	1.48E-10	1.30E-09	*ns*			**8.8**	6.54E-27	1.69E-25	**2.3**	3.47E-06	2.52E-05
Plat	plasminogen activator, tissue	*ns*			*ns*			**3.7**	9.75E-70	8.84E-68	**4.0**	7.37E-127	3.92E-124
Plau	plasminogen activator, urokinase	*ns*			*ns*			**3.73**	2.31E-129	5.90E-127	*ns*		
Pdgfrb	platelet derived growth factor receptor, beta polypeptide	**-5.5**	1.78E-247	8.15E-245	*ns*			**3.7**	6.91E-150	2.37E-147	*ns*		
Pdgfd	platelet-derived growth factor, D polypeptide	**-14.5**	3.91E-149	6.98E-147	*ns*			**3.7**	1.97E-90	2.54E-88	*ns*		
Sema6d	sema domain, transmembran	*ns*			*ns*			**3.7**	5.36E-97	7.76E-95	**2.34**	7.77E-69	1.22E-66

	e domain (TM), and cytoplasmic domain, (semaphorin) 6D												
Slit2	slit homolog 2 (Drosophila)	*ns*			*ns*			**3.7**	5.11E-55	3.31E-53	*ns*		

Table S16

TMM for the DEGs related to *Proliferation* depicted in Fig. 5d.

Gene	NAME	WT CTRL 1	WT CTRL 2	WT CTRL 3	siCOUP-TFII WT 1	siCOUP-TFII WT 2	siCOUP-TFII WT 3	THRA-PV CTRL 1	THRA-PV CTRL 2	THRA-PV CTRL 3	siCOUP-TFII THRA-PV 1	siCOUP-TFII THRA-PV 2	siCOUP-TFII THRA-PV 3
Ccl5	chemokine (C-C motif) ligand 5	0.00	0.32	0.15	0.72	0.55	0.66	3.62	1.78	2.93	18.35	20.22	14.69
Cnn1	calponin 1	132.8	119.7	114.7	63.7	67.6	70.9	16.2	11.4	18.8	70.5	78.2	80.6
Ereg	epiregulin	65.1	66.7	63.9	48.7	43.3	41.8	7.2	7.0	8.9	24.2	21.7	20.7
Foxc2	forkhead box C2	54.4	55.3	54.0	49.3	60.9	55.6	15.3	15.8	16.9	42.7	39.7	44.2
Gata6	GATA binding protein 6	6.9	6.4	6.2	6.9	6.6	5.8	0.7	0.4	1.2	4.2	4.4	4.9
Htr1b	5-hydroxytryptamine (serotonin) receptor 1B	26.8	26.3	27.1	23.6	22.4	26.8	5.5	8.3	6.9	19.4	18.9	17.6
Igfbp5	insulin-like growth factor binding protein 5	225	218	230	376	403	416	89	101	107	1453	1213	1674
Il6	interleukin 6	6.3	6.6	6.9	6.0	4.1	4.7	0.4	0.1	0.8	3.3	2.8	4.8
Myocd	myocardin	1.1	0.9	1.1	1.2	1.0	1.5	0.9	0.5	0.5	3.2	2.7	2.3
Ndrg4	N-myc downstream regulated gene 4	61.1	61.1	57.2	58.3	58.8	56.4	19.1	22.9	24.1	61.1	55.4	58.4
Nog	noggin	3.0	3.2	2.4	5.1	4.7	4.5	1.9	1.6	2.9	11.3	10.1	11.0
Nr4a3	nuclear receptor subfamily 4, group A, member 3	2.8	2.6	3.4	4.5	5.9	5.3	1.3	0.4	0.6	5.1	7.5	7.6
Pdgfd	platelet-derived growth factor, D polypeptide	20.8	23.8	22.5	21.8	19.0	21.2	1.3	1.8	1.4	11.2	13.0	14.5
Pdgfrb	platelet derived growth factor receptor, beta polypeptide	333	334	303	321	332	315	59	59	59	230	193	228
Pparg	peroxisome proliferator activated receptor gamma	14.7	13.9	14.5	28.6	32.6	29.0	1.9	2.6	3.2	29.7	35.1	36.7
Ptgs2	prostaglandin-endoperoxide synthase 2	1292	1314	1286	1695	1520	1457	129	127	141	573	534	622

Smpd3	sphingomyelin phosphodiesterase 3, neutral	80.9	79.6	71.3	57.3	57.7	55.3	5.3	5.6	5.7	23.1	20.4	25.7
Tbx2	T-box 2	3.6	3.7	3.6	5.6	6.6	5.0	3.5	1.6	3.6	10.8	12.0	15.6
Tgm2	transglutaminase 2, C polypeptide	109	108	99	75	75	78	7	11	11	26	29	37

Table S17

Fold change (FC), p value and (False Discovery Rate (FDR) for the DEGs related to *Muscle Cell Proliferation* depicted in Fig. 4D.

		**TRα1PV vs WT**			**siCOUP-TFII WT vs WT**			**siCOUP-TFII TRα1PV vs TRα1PV**			**siCOUP-TFII TRα1PV vs WT**		
**Gene**	**NAME**	**FC**	**p Value**	**FDR**	**FC**	**p Value**	**FDR**	**FC**	**p Value**	**FDR**	**FC**	**p Value**	**FDR**
Ccl5	chemokine (C-C motif) ligand 5	**17.3**	4.22E-26	9.13E-25	*ns*			**6.4**	6.51E-31	2.05E-29	**110.5**	7.36E-85	1.83E-82
Cnn1	calponin 1	**-7.9**	1.34E-147	2.37E-145	*ns*			**4.9**	8.52E-92	1.13E-89	*ns*		
Ereg	epiregulin	**-8.5**	7.97E-244	3.52E-241	*ns*			**2.9**	5.45E-57	3.75E-55	**-2.9**	2.45E-80	5.49E-78
Foxc2	forkhead box C2	**-3.4**	5.09E-107	5.83E-105	*ns*			**2.6**	8.75E-68	7.52E-66	*ns*		
Gata6	GATA binding protein 6	**-8.2**	4.75E-40	1.61E-38	*ns*			**5.6**	6.28E-27	1.63E-25	*ns*		
Htr1b	5-hydroxytryptamine (serotonin) receptor 1B	**-3.9**	1.64E-66	1.02E-64	*ns*			**2.7**	2.59E-36	9.81E-35	*ns*		
Igfbp5	insulin-like growth factor binding protein 5	**-2.3**	7.87E-34	2.24E-32	*ns*			**14.6**	2.26E-289	9.80E-286	**6.4**	1.04E-156	9.99E-154
Il6	interleukin 6	**-15.8**	4.04E-34	1.16E-32	*ns*			**8.7**	3.78E-21	7.27E-20	*ns*		
Myocd	myocardin	*ns*			*ns*			**4.3**	5.72E-11	5.58E-10	**2.6**	6.74E-06	4.68E-05
Ndrg4	N-myc downstream regulated gene 4	**-2.7**	2.83E-84	2.34E-82	*ns*			**2.6**	8.89E-82	9.97E-80	*ns*		
Nog	noggin	*ns*			*ns*			**5.0**	5.61E-35	2.01E-33	**3.8**	1.82E-24	5.72E-23
Nr4a3	nuclear receptor subfamily 4, group A, member 3	**-3.9**	1.48E-10	1.30E-09	*ns*			**8.8**	6.54E-27	1.69E-25	**2.3**	3.47E-06	2.52E-05
Pdgfd	platelet-derived growth factor, D polypeptide	**-14.5**	3.91E-149	6.98E-147	*ns*			**3.7**	1.97E-90	2.54E-88	*ns*		
Pdgfrb	platelet derived	**-5.5**	1.78E-247	8.15E-245	*ns*			**3.7**	6.91E-150	2.37E-147	*ns*		

	growth factor receptor, beta polypeptide												
Pparg	peroxisome proliferator activated receptor gamma	**-5.5**	1.22E-72	8.40E-71	**2.1**	3.63E-23	7.34E-21	**3.7**	1.95E-181	1.21E-178	**2.4**	2.26E-31	9.74E-30
Ptgs2	prostaglandin-endoperoxide synthase 2	**-9.8**	0	0	*ns*			**3.7**	8.21E-164	3.68E-161	**-2.2**	4.53E-55	4.87E-53
Smpd3	sphingomyelin phosphodiesterase 3, neutral	**-13.9**	0	0	*ns*			**3.7**	4.71E-97	6.86E-95	**-3.3**	1.66E-108	6.08E-106
Tbx2	T-box 2	*ns*			*ns*			**4.4**	9.57E-21	1.80E-19	**3.5**	1.04E-15	2.01E-14
Tgm2	transglutaminase 2, C polypeptide	**-10.7**	1.74E-165	3.82E-163	*ns*			**3.7**	6.82E-41	2.99E-39	**-3.4**	1.05E-55	1.17E-53

Supplementary Materials

List of antibodies used in Immunofluorescence and Western Blot

Antibody (host species)	Application	Dilution	Cat #	Company
**Anti- Myosin Heavy Chain (MYH1E) (m)**	Immunofluorescence	1:100	MF20	DSHB
**Anti-Laminin (r)**	Immunofluorescence	1:100	ab11575	Abcam
**Anti-COUP-TFII (m)**	Immunofluorescence	1:100	PP-H7147-00	R&D Systems
**Donkey anti-Rabbit IgG (H+L) Highly Cross-Adsorbed Secondary Antibody, Alexa Fluor 555**	Immunofluorescence	1:300	A31572	Life Technology
**Goat anti-Rabbit IgG (H+L) Cross-Adsorbed Secondary Antibody, Alexa Fluor 555**	Immunofluorescence	1:300	A21428	Life Technology
**COUP-TFII (D16C4) (r)**	Western Blot and CoIp	1:1000	6434	Cell Signaling
**Anti-Thyroid Hormone Receptor α (r)**	Western Blot	1:1000	SAB4502968	Sigma-Aldrich
**Anti-Histone H3 (1H3) (r)**	Western Blot	1:1000	9717	Cell Signaling
**Anti-B actin (r)**	Western Blot	1:1000	GTX109639	Gene Tex
**Anti-Rabbit IgG (whole molecule)–Peroxidase antibody produced in goat**	Western Blot	1:35000	A9169	Sigma-Aldrich
**Rabbit Anti-Mouse IgG Antibody, HRP conjugate**	Western Blot	1:40000	AP160P	Millipore
**VeriBlot for IP Detection Reagent (HRP)**	Western Blot (CoIP)	1:2000	ab131366	Abcam
